# Transfusion-transmitted arboviruses: Update and systematic review

**DOI:** 10.1371/journal.pntd.0010843

**Published:** 2022-10-06

**Authors:** Ángel Giménez-Richarte, María Isabel Ortiz de Salazar, María-Paz Giménez-Richarte, Miriam Collado, Pedro Luís Fernández, Carlos Clavijo, Laura Navarro, Cristina Arbona, Pascual Marco, Jose-Manuel Ramos-Rincon

**Affiliations:** 1 Valencian Community Blood Transfusion Center, Valencia, Spain; 2 Medicial student, Miguel Hernandez University of Elche, Alicante, Spain; 3 Service of Hematology, General- University Hospital of Alicante-ISABIAL. Alicante, Spain; 4 Clinical Medicine Department, Miguel Hernandez University of Elche, Alicante, Spain; Universite de Montreal, CANADA

## Abstract

**Background:**

The detection of the first cases of transfusion-transmitted West Nile virus in 2002 posed a new challenge for transfusion safety. Institutions like the World Health Organization have stated that blood transfusion centers need to know the epidemiology of the different emerging infectious agents and their impact on blood transfusion. The aim of the study is to review the published cases of arbovirus transmission through transfusion of blood or blood components and to analyze their main clinical and epidemiological characteristics.

**Material and methods:**

Systematic literature searches were conducted in MEDLINE, Embase and Scopus. Pairs of review authors selected a variety of scientific publications reporting cases of transfusion-transmitted arboviruses. Main clinical and epidemiological characteristics were reviewed of the cases described. The study protocol was registered in PROSPERO CRD42021270355.

**Results:**

A total of 74 cases of transfusion-transmitted infections were identified from 10 arboviruses: West Nile virus (n = 42), dengue virus (n = 18), Zika virus (n = 3), yellow fever vaccine virus (n = 3), tick-borne encephalitis virus (n = 2), Japanese encephalitis virus (n = 2), Powassan virus (n = 1), St. Louis encephalitis virus (n = 1), Ross River virus (n = 1) and Colorado tick fever virus (n = 1). The blood component most commonly involved was red blood cells (N = 35, 47.3%; 95% confidence interval [CI] 35.9% to 58.7%). In 54.1% (N = 40; 95% CI: 42.7%-65.47%) of the cases, the recipient was immunosuppressed. Transmission resulted in death in 18.9% (N = 14; 95% CI: 10.0%-27.8%) of the recipients. In addition, 18 additional arboviruses were identified with a potential threat to transfusion safety.

**Discussion:**

In the last 20 years, the number of published cases of transfusion-transmitted arboviruses increased notably, implicating new arboviruses. In addition, a significant number of arboviruses that may pose a threat to transfusion safety were detected. In the coming years, it is expected that transmission of arboviruses will continue to expand globally. It is therefore essential that all responsible agencies prepare for this potential threat to transfusion safety.

## Author summary

The transfusion of blood and blood components entails some risks and potential complications, chief among them the transmission of infectious agents. Organizations like the American Association of Blood Banks have warned of the risks posed by emerging and re-emerging viruses for transfusion safety and have classified transmission of several arboviruses as a high or very high risk to the transfusion of blood and blood components. Following recommendations by the World Health Organization (WHO), this study aims to enable safe blood transfusion services, by making available the latest updated information of transfusion-transmitted arboviruses and comprehensive knowledge of the current epidemiology of reported cases.

Research revealed case reports of transfusion transmission of 10 arboviruses, with West Nile virus and dengue virus as the most prevalent. Main clinical characteristics of reported cases were collected, including the type of blood component transfused and the state of immunosuppression of the recipient. Research also revealed 18 additional arboviruses with potential risk of transmission through other direct transmission routes. This systematic review provides an updated overview of the clinical characteristics of reported cases of transfusion-transmitted arboviruses. It is the most complete record published to date that assesses the risk posed by arboviruses to blood transfusion.

## Background

Throughout history, transfusion safety has been threatened by the emergence of various infectious agents [1]. Currently, health service consumers expect a total absence of transfusion-related transmission of infectious diseases. However, the 2002 notification of the first cases of West Nile virus transmission through transfusion of blood components, highlighted the threat posed to transfusion safety by emerging and re-emerging infectious agents [2,3].

Among the emerging viruses, arboviruses are especially relevant due to their known or theoretical potential for transmission through blood transfusion. These viruses are transmitted by arthropods, mainly mosquitoes (genera *Culex* spp. and *Aedes* spp.) and ticks (particularly the genus *Ixodes*) but also flies, dragonflies, and lice. In humans, they can cause clinical presentations ranging from asymptomatic infections or mild flu-like symptoms, to severe, potentially fatal hemorrhagic and neurological syndromes. Their epidemiology is highly variable and unpredictable and may manifest as isolated and localized cases; seasonal waves; or widespread, epidemic outbreaks. Originally from tropical regions, arboviruses have now spread widely, producing numerous epidemic outbreaks over the past 20 years and constituting a major public health problem all over the planet. Arboviruses are classified into different taxonomic families: *Flaviviridae*, *Bunyaviridae*, *Togaviridae*, *Rhabdoviridae*, *Reoviridae*, and *Asfarviridae*. The most important arboviruses that cause disease in humans belong to the families *Flaviviridae*, *Togaviridae* and *Bunyaviridae* [4].

Threats to transfusion safety arising from new infectious agents have traditionally been addressed through the development of methods to screen blood donors, and this also holds true for arboviruses. Screening methods used include serological and non-serological antigen rapid testing techniques as well as nucleic acid (PCR) tests. These tests may be used routinely or according to the epidemiological profile of the areas or countries in question. However, they may not be sufficient to fully guarantee transfusion safety in the case of donors with a very low viral load and/or who are in the window period after infection [5].

In recent years, various international organizations have created tools to assess the transfusion risk posed by these newly emerging and re-emerging infectious agents. In 2009, the AABB Transfusion-Transmitted Diseases Committee, in collaboration with members of the Food and Drug Administration (FDA), the Centers for Disease Control and Prevention (CDC), and other agencies, developed guidance to identify infectious agents that could pose a real or potential risk to transfusion safety. In this process, 68 infectious agents, including arboviruses, were classified according to their epidemiological profile and the public’s awareness and perception of them. Of the arboviruses in this classification, dengue virus was assigned in the highest risk category, while chikungunya and St. Louis encephalitis viruses were included in the second-highest risk category [6]. In 2011, fact sheets were developed for miscellaneous arboviruses, including for yellow fever virus and yellow fever vaccine virus [7]. Various infectious agents were also classified in 2011 by the European Center for Disease Prevention and Control (ECDC) based on the epidemiological and environmental characteristics of these viruses in Europe. The agents given the highest priority include a number of arboviruses: West Nile virus, dengue virus, chikungunya virus and tick-borne encephalitis virus [8]. In Asia, the WHO also published the *Asia Pacific Strategy for Emerging Diseases and Public Health Emergencies* (APSED III) in 2017 with the aim of detecting and addressing the different emerging infectious diseases in the region [9].

With the development of these risk assessment tools, major global public health entities have expressed the need for comprehensive knowledge of the epidemiological characteristics of the various emerging transfusion-transmitted infections and to evaluate their potential impact on donor selection criteria and the supply of blood and blood products [10].

The main objective of this study is, therefore, to review the published cases of arbovirus transmission through the transfusion of blood or blood components throughout the history of transfusion and to study their main clinical and epidemiological characteristics. For the research question in PICOT format, please see (S1 Table). The secondary objective is to, additionally, identify arboviruses that can be a threat to transfusion safety through other non-vector, non blood transfusion related direct routes of transmission such as: organ and/or hematopoietic stem cell transplantation, mother-to-child transmission, direct blood contact or by their prevalence in blood donors.

## Materials and methods

### Design

This systematic literature review was designed and conducted in accordance with the *Cochrane Handbook of Systematic Reviews of Interventions* and reported in line with the Preferred Reporting Items for Systematic Reviews and Meta-Analyses (PRISMA) Statement [11]. For the PRISMA checklist, please see the (S1 PRISMA Checklist). The protocol for this systematic review was published and registered in PROSPERO (www.crd.york.ac.uk/prospero, CRD42021270355).

First, a global descriptive analysis was completed including all cases of transfusion-transmitted arboviruses. Consequently, a brief study was conducted of the main clinical and epidemiological characteristics of reported cases of arboviral transfusion transmission. After a general analysis that included all cases, we specifically analyzed the arboviruses with the most reported cases of transmission through blood transfusion or blood components: West Nile virus and dengue virus. Finally, a descriptive analysis was performed of arboviruses that can be considered a potential threat to transfusion safety, based on published reports of any non-vector, non-blood related direct route of transmission or because of their prevalence in blood donors, regardless of reported evidence of transfusion transmission.

### Source of data collection

Systematic literature searches were conducted in MEDLINE, Embase, Scopus using the server of University Miguel Hernández server.

### Search strategy

The complete search strategy is described in more detail in the study protocol and (S1 Text). Initially, the authors have performed searches with free text terms in all three bibliographic databases using the term "transfusion" in combination with the name of the arboviruses being reviewed. In MEDLINE, Medical Subject Headings (MeSH) such as “Blood Transfusion”[Mesh] OR “Transfusion Reaction”[Mesh] also served as search terms. In Embase the Entree thesaurus was used to search articles based on the following terms: “blood transfusion”/exp.

As a second search strategy, free text searches were performed in MEDLINE using the terms: “transplantation”, “vertical transmission”, “blood donors”, while in Embase a free text search was conducted by entering the following terms in the Entree Thesaurus: “transplantation”/exp OR transplantation, “vertical transmission”/exp OR “vertical transmission” AND “blood donor”/exp OR “blood donor”. In Scopus, we ran searches only using free text terms in the fields for title, abstract, and key words. Bibliographies of all included records to identify other potentially relevant publications were also hand-searched.

The authors included records published from the year of inception of each bibliographic database (start date—we did not apply any specific limits to the start date) to 10 November 2021 (end date). The records were entered into the Mendeley Desktop reference manager (Elsevier).

### Study selection

In answer to the primary objective, selected publications were those in any language describing cases of transfusion-transmitted arboviruses transmitted through transfusion of blood or blood components. A wide variety of scientific studies (original articles, reviews, brief reports, case reports, letters to the editor, conference papers and others) were included reporting cases of arboviruses transmitted through transfusion of blood or blood components. Publications that collected cases of transmission in non-humans were excluded, as were publications that did not detail the specific type of arbovirus transmitted through the transfusion of blood or blood components.

Search records were independently evaluated by two review authors for inclusion in the publication overview. Any discrepancies were resolved by consensus. Inter-observer agreement was measured by determining the kappa index.

The bibliographic reference manager Mendeley Desktop was used to create folders containing records for each virus, eliminating duplicates. Titles and abstracts were screened to pre-select records in any language that included general information on the possible transmission of arboviruses through the transfusion of blood or blood components. Consequently, full texts of all potentially relevant records were reviewed independently by two researchers. Records included, were those that reported cases of transfusion transmission of blood or blood components in humans over the entire period under study.

To answer to the secondary objective, publications in any language were first selected, describing cases stemming from direct non-vector or transfusion related routes of arbovirus transmission (e.g.: organ and/or hematopoietic stem cell transplantation, mother-to-child transmission, direct blood contact) or reports of prevalence of these arboviruses in blood donors. Consequently, a wide variety of records (original articles, reviews, brief reports, case reports, letters to the editor, conference papers and others) were included to accommodate this review. Lastly, for each encountered route of transmission and each arbovirus found prevalent in blood donors, one publication was selected for the produced publication overview, representing the routes of transmission or evidence of prevalence in screened blood donors. Representative publications were selected based on the following criteria: most recent date of publication, most cited publication, or publication in a journal with the highest impact factor. This evaluation was independently done by two review authors. Final selection for inclusion in the publication overview was determined by consensus. Inter-observer agreement was measured by determining the kappa index.

### Data extraction and analysis

All data of cases of transfusion-transmitted arboviruses were extracted from the selected publications and directly entered into a Microsoft Excel database by a single review author. Data quality control was consequently done by a second review author. If a publication included more than one case of transfusion transmission, each case was recorded and entered separately in the database. Basic epidemiological and clinical data of the reported cases of transfusion transmission were then analyzed: type of blood component implicated, immunosuppression and clinical presentation in the recipient, fatality of the outcome following transmission as well as the plausibility of the respective transmission route. From the selected publications, study authors conclusions on transfusion transmission and mortality, attributable to the arboviral infection, were recorded. Determinants of potential alternative routes of transmission were additionally collected such as: exposure to the vector, prolonged hospital stay, additional patients infected by the same contaminated donated product or whether the authors themselves ruled out other potential explanations for the patient’s infection.

Results were expressed as proportion with a 95% confidence interval (95% CI). The proportion was calculated as the number of the reported parameter under study divided by the total number of reported cases of transfusion transmission (Example: number of cases of transfusion-transmitted arboviruses related with the transfusion of red blood cells / total number of cases of transfusion-transmitted arboviruses x 100). The 95% CIs were for proportions (p±Z_α/2_√(p(1-p)/n)).

### Quality assessment

Due to the mainly descriptive objective of this study and the type of publications included (case reports, letters to the editor), no standard quality control tool, such as ROBINS-I or STROBE checklist, was applied for assessing risk of bias in non-randomized studies.

## Results

**Fig 1:**
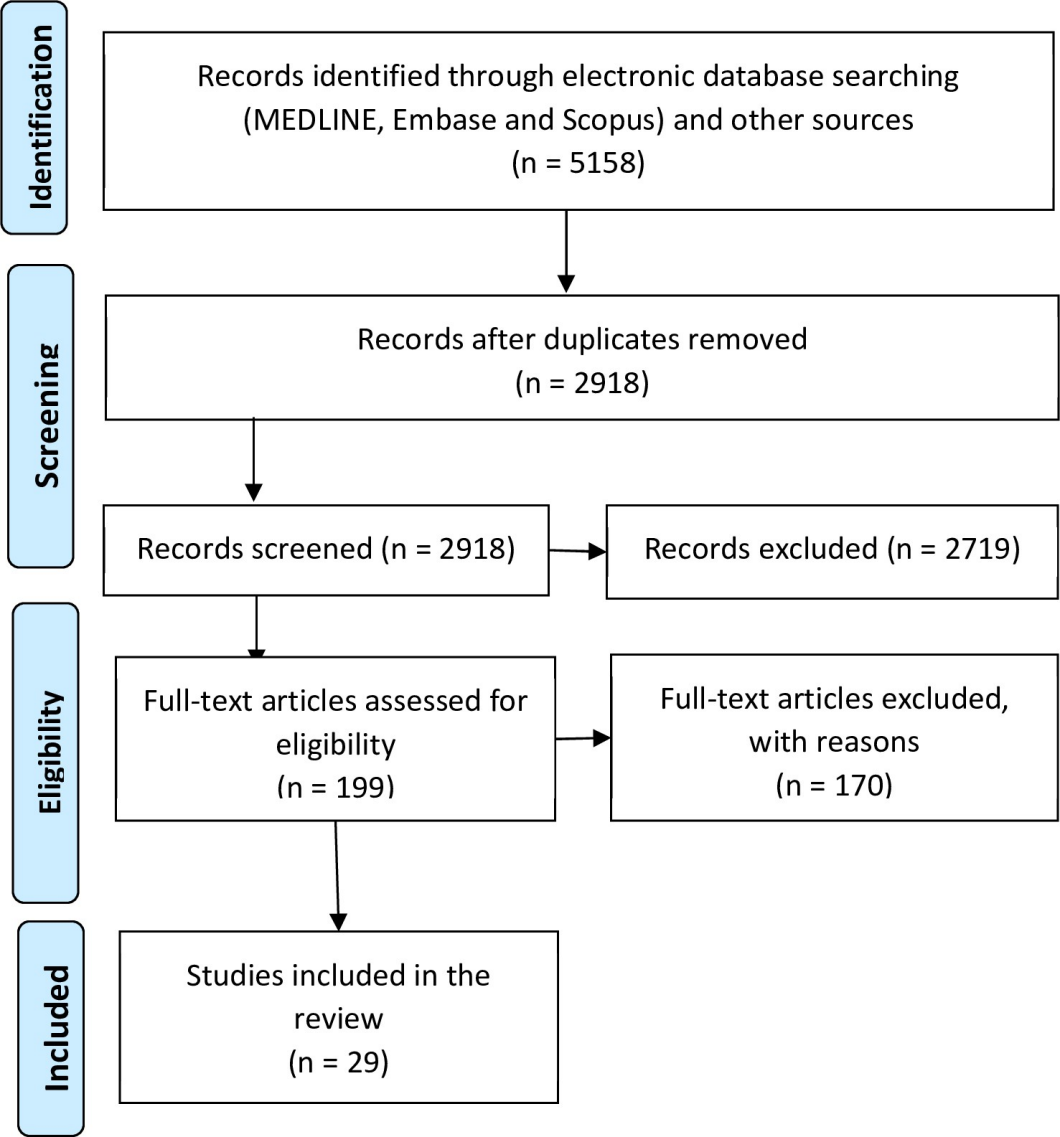
PRISMA flow chart on selection of included studies covered by the literature review.

The search strategy yielded a total of 5158 records. After eliminating duplicates, the authors screened the title and abstract of 2918 records and retrieved the full text of 199. The authors selected any records that suggest human transmission of arboviruses through blood transfusion or studies that display information that could facilitate the identification of other publications that report cases of transfusional transmission of arboviruses. Finally, 29 studies reporting cases of human transmission of arboviruses through transfusion of blood or blood components were included. Fig 1 presents the PRISMA flow chart, describing the selection process for including published studies of transfusion-transmitted arboviruses.

Of the 29 records included, 11 studies described transfusion-transmitted West Nile virus; 9, dengue virus; 2, Zika virus; 1 Japanese encephalitis virus; 1, St. Louis encephalitis virus; 1, tick-borne encephalitis virus; 1, Powassan virus; 1, yellow fever virus; 1, Colorado tick fever virus; and 1, Ross River fever virus. Interobserver concordance was very high (κ = 0.97). For the PRISMA flow chart assessing each specific arbovirus, please see the (S1 Flow Chart).

For the PRISMA flow chart with the process of selecting records describing non-vector and non-transfusion related direct routes of transmission of arboviruses and publications reporting the prevalence of arboviruses in blood donors, please see (S2 Flow Chart). A total of 32 records were selected for the publication overview with an interobserver K value of 0.84.

### Analysis of results

**Fig 2:**
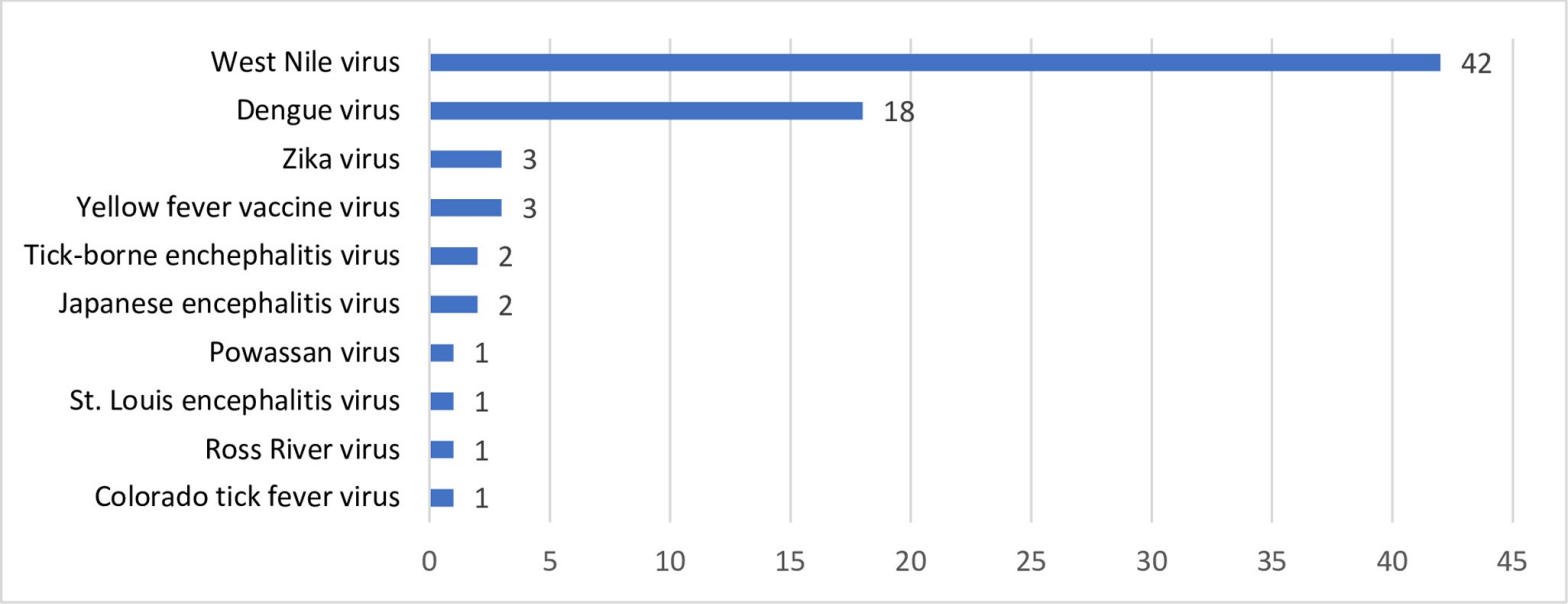
Reported transfusion-transmitted arbovirus cases.

Included studies described 74 cases of suspected transfusion transmission of 10 arboviruses. Most cases involved transfusion transmission of West Nile virus (N = 42, 56.8%; 95% CI: 45.5–68.0%) and dengue virus (N = 18, 24.3%; 95% CI: 14.5–34.1%). Remaining cases described transfusion transmission of Zika virus, yellow Fever vaccine virus, tick-borne encephalitis virus, Japanese encephalitis virus, Powassan virus, St. Louis encephalitis virus, Ross River virus and Colorado tick fever virus (Fig 2).

The USA was the country with the most reported cases of transfusion transmission (N = 42, 62%.2%, 95% CI: 51.1–73,2%). The main type of blood component implicated was red blood cells (N = 35, 47.3%; 95% CI: 35.9–58.7%).

**Fig 3:**
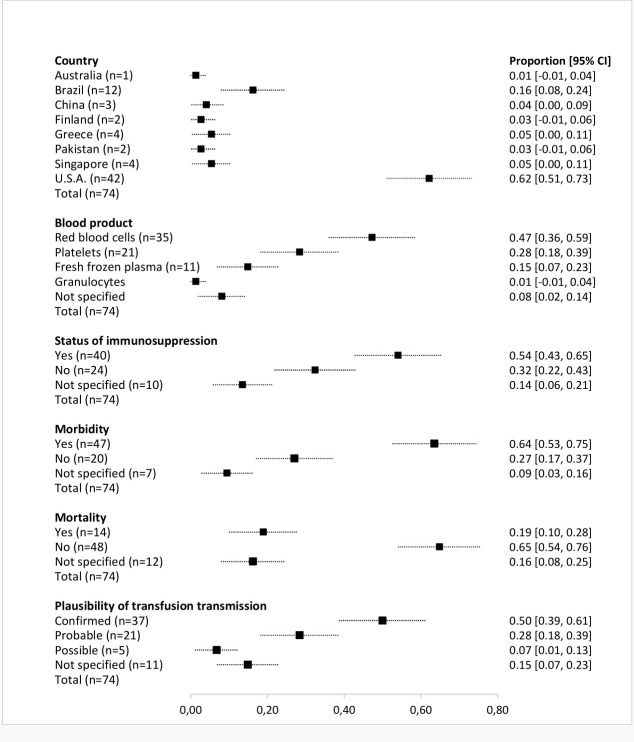
Main clinical and epidemiological characteristics of reported cases of transfusion-transmitted arboviruses.

Over half of the reported transfusion-transmitted arbovirus cases, selected for the overview, were described in recipients classified as immunocompromised (N = 40, 54.1%; 95% CI: 42.7–65.5%). Arboviral morbidity and mortality was reported in 63.5% (N = 47; 95% CI: 52.5–74.5%) and 18.9% (N = 14; 95% CI: 10.0–27.8%) of cases described, respectively. Recipient mortality was mostly reported for the West Nile virus (N = 13; 92.9%; 95% CI: 79.4–100%) and 78.6% (N = 13; 95% CI: 57.1–100%). of fatal cases the recipient was classified as immunocompromised. Plausibility of transfusion-transmission was confirmed by the authors of the selected records in 50.0% (N = 37; 95% CI: 38.6–61.4%) of cases. For the main clinical and epidemiological characteristics of selected cases, see Fig 3.

**Fig 4:**
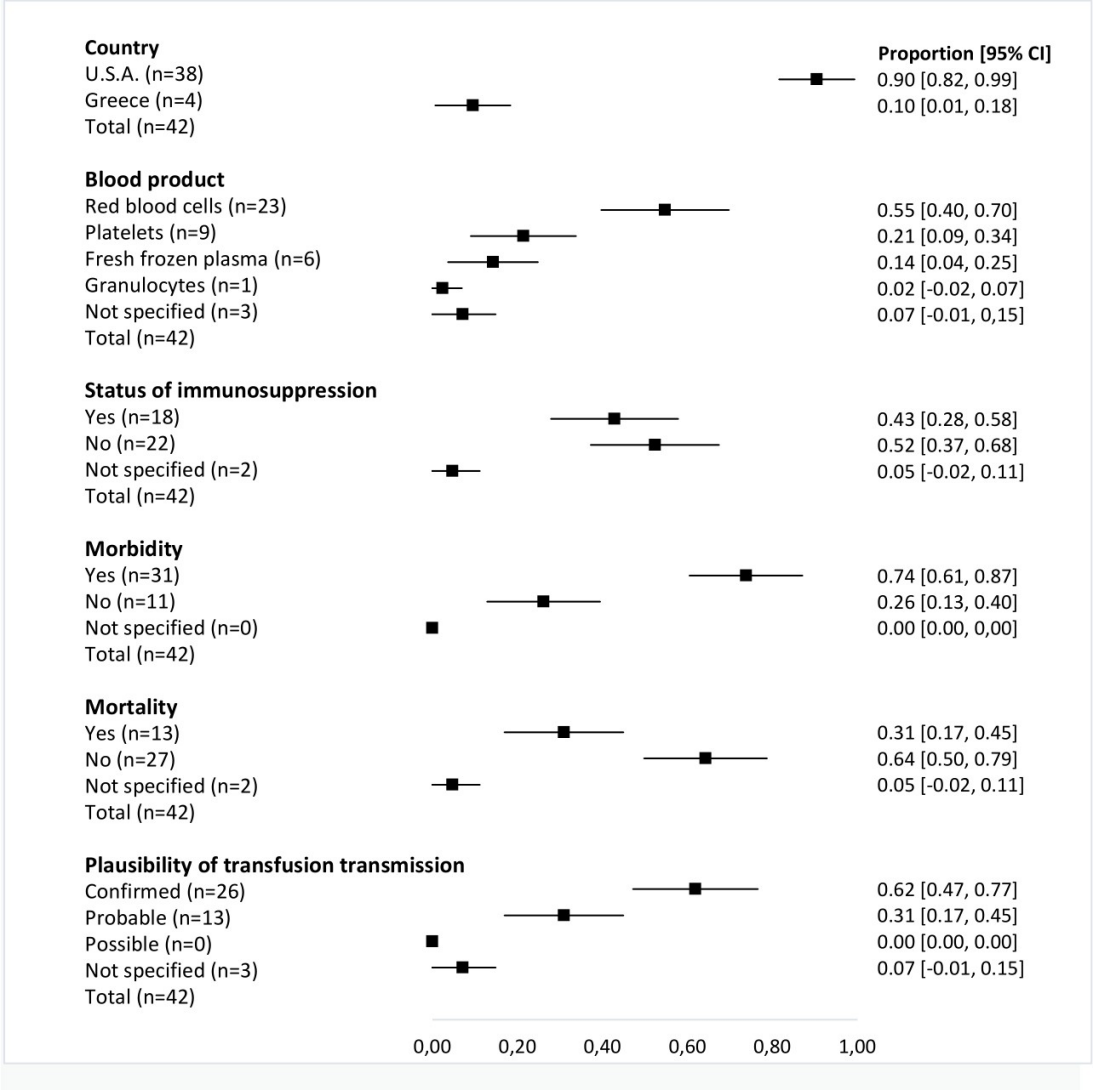
Main reported clinical and epidemiological characteristics of transfusion-transmitted West Nile virus.

Regarding West Nile virus, 90.5% (N = 38; 95% CI: 81.6–99.4%) of reported cases were diagnosed in the USA, and the main type of blood component implicated was red blood cells 54.8% (N = 23; 95% CI: 39.7–69.8%). Symptoms as a result of transmission were reported in 73.8% (N = 31; 95% CI: 60.5–87.1%) of recipients and in 31.0% (N = 13; 95% CI: 17.0–44.9%) death was reported as the ultimate outcome of transmission. Fig 4 shows the main reported clinical and epidemiological characteristics of cases of West Nile virus transfusion transmission.

**Fig 5:**
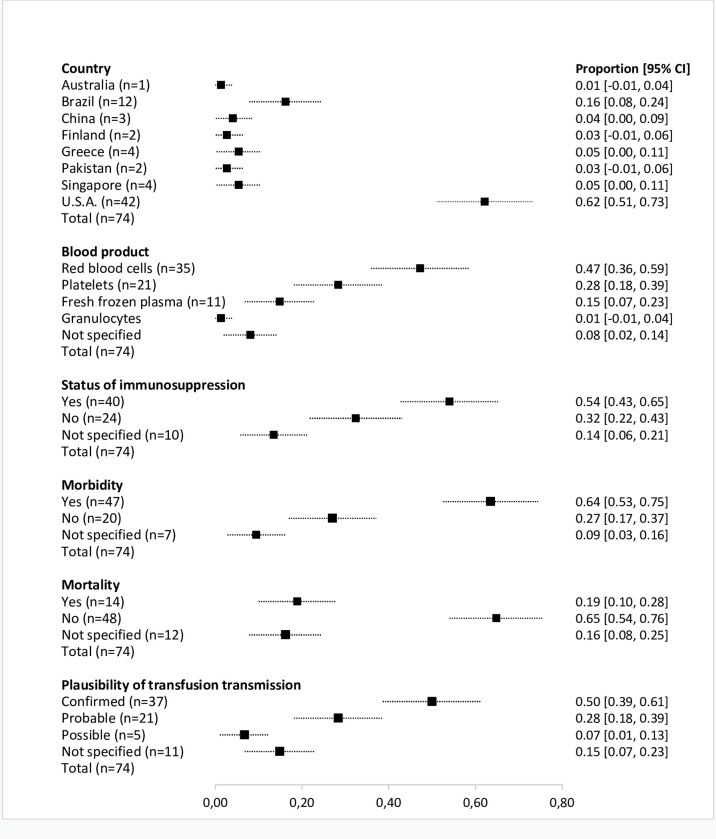
Main reported clinical and epidemiological characteristics of transfusion-transmitted dengue virus.

Of the reported transfusion transmitted cases related to dengue virus, 50% (N = 9; 95% CI: 26.9–73.1%) originated from Brazil. Transfusion of red blood cells was the main route of transmission described (N = 8, 44.4%; 95% CI: 21.5–67.4%). Symptoms were reported for 61.1% (N = 11; 95% CI: 38.6–83.6%) of recipients. None of the selected cases of transfusion transmission of dengue virus were reported as fatal. In 38.9% (N = 7; 95% CI: 16.4–61.4%) of cases, the serotype involved was DENV-4. Fig 5 presents the main reported clinical and epidemiological characteristics of cases of transfusion transmission of dengue virus.

Tables 1, 2 and 3 provide an overview of the main the main characteristics of reported cases of transfusion-transmitted arboviruses apart from dengue and West Nile virus.

**Table 1:** Transfusion-transmitted arboviruses cases.

Virus	Publication	Type of publication	Country	Year	Blood product	Recipient	IC	Morbidity	Mortality	Diagnostic method	Alternative explanation						Other non-vector, non-blood transfusion related transmission
											*Exposure to Vector*	*Prolonged hospital stay*	*Other infected recipients*	*Non-vector vias rejected*	Plausibility	Additional data	
**Colorado tick fever virus**	Randall 1975 [12]	Case report	U.S.A.	1975	Not specified (WB?)	Colon carcinoma	Yes	Yes	No	Clinical Not specified	No	−	−	−	−	−	Vertical transmission[13]
**Japanese encephalitis virus**	Cheng 2018 [14]	Original article	China	2017	RBC	Double lung transplantation	Yes	Yes	Yes	RT-PCR Sanger sequencing Phylogenetic analysis	N.P.	Yes	Yes	Yes	Confirmed	Same donor No data available about PL recipient	Screening in blood donors[15] Vertical transmission[12]
					Platelets	Acute myeloid leukemia	Yes	No	No	Serology IgM	−	−	Yes	−			
**Ross River virus**	Hoad 2015 [16]	Case reports	Australia	2014	RBC	Myelodysplastic syndrome	Yes	No	No	Serology IgM RT-PCR	N.P.	−	No	−	−	−	Screening in blood donors[17] Vertical transmission[12]
**St. Louis encephalitis virus**	Venkat 2017 [18]	Original article	U.S.A.	2015	RBC	Kidney transplant	Yes	Yes	−	Serology IgM+ PRNT	N.P.	Yes	No	Yes	Possible	No data available about PL recipient.	Screening in blood donors[19]
**Powassan virus**	Taylor 2020 [20]	Original article	U.S.A.	2018	RBC	Kidney transplant	Yes	Yes	No	Serology IgM+ PRNT RT-PCR	No	Yes	No *	Yes	Probable	−	−
**Tick-borne encephalitis virus**	Wahlberg 1989 [21]	Original article	Finland	1959–1987	Not specified	Not specified	−	Yes	No	Not specified	N.P.	−	−	−	−	−	Blood contact[22] S.O.T. transmission[23] Screening in blood donors[24]
					Not specified	Not specified	−	Yes	No	Not specified	N.P.	−	−	−	−		
**Yellow fever vaccine virus**	Lederman 2010 [25]	Original article	U.S.A.	2009	Platelets (irradiated)	Wilm’s tumor (relapsed), QMT	Yes	No	No	Serology IgM+ PRNT	−	−	−	−	−	2 exposed recipients without evidence of transmission	Screening in blood donors[26] Vertical transmission[12]
					Platelets	Kidney/liver transplantation, DM	Yes	No	No		−	−	−	−	−		
					FFP (2 units)	CKF, TBC, psoriasis (infliximab)	Yes	No	No		−	−	−	−	−		
**Zika virus**	Barjas-Castro 2016 [27]	Case report	Brazil	2015	Platelets (Pool)	Liver transplantation	Yes	No	No	Virus isolation RT-PCR Phylogenetic analysis	No	No	No *	−	Probable		Blood contact[29] Screening in blood donors[30] Vertical transmission[31]
	Motta 2016 [28]	Letter to the editor	Brazil	2016	Platelets (apheresis)	Primary myelofibrosis	Yes	No	No	Serology IgG/IgM+ PRNT RT-PCR Molecular sequencing Phylogenetic analysis	−	−	Yes	−	Confirmed	Same donor	
					Platelets (apheresis)	Acute myeloid leukemia—BMT	Yes	No	No		No	Yes	Yes	−			

BMT: bone marrow transplantation, CKD: chronic kidney disease, CTX: chemotherapy, DM: diabetes mellitus, FFP Fresh frozen plasma, NP: not probable, PL: plasma, PRNT Plaque reduction neutralization test, TBC Tuberculosis, RBC red blood cells, IC: Immunocompromised, RT-PCR Reverse transcription polymerase chain reaction, SOT: Solid organ transplantation, ZIKV: Zika virus, RNA: Ribonucleic Acid

− Data not available

* PL not transfused was positive

**Table 2:** Transfusion-transmitted dengue virus cases.

Publication	Type of publication	Country	Year	Blood product	Recipient	IC.	Morbidity	Mortality	Diagnostic method	Serotype	Alternative explanation				Plausibility	Additional data	Other non-vector, non-blood transfusion related transmission
											*Exposure to Vector*	*Prolonged hospital stay*	*Other infected recipients*	*Non-vector vias rejected*			
Chuang 2008 [32]	Original article	China	2002	RBC	Bronchiectasis, vitamin B12 deficiency anaemia	No	Yes	No	Serology IgM+ HI RT-PCR	1	−	−	−	−	Confirmed		
Tambyah 2008 [33]	Letter to the editor	Singapore	2007	RBC	DM, ischemic heart disease	Yes	Yes	No	Serology IgG/IgM PCR Sequencing	2	−	−	Yes	−	−	Same donor	Blood contact (needle-stick related transmissión) [41] HSCT transmission [42] Screening in blood donors [43] S.O.T. transmission [44] Vertical transmission [45]
				Platelets	Hepatocellular carcinoma	Yes	No	No			−	−	Yes	−	−		
				FFP	DM, hypertension, ischemic heart disease, CRI.	Yes	Yes	No			N.P.	Yes	Yes	−	−		
Boon Oh 2015 [34]	Case report	Singapore	Not specified, 2014?	RBC	Gastrointestinal bleeding.	No	Yes	No	Serology IgG/IgM RT-PCR Sanger sequencing	2	No	Yes	No *	−	Confirmed		
Levi 2015 [35]	Original article	Brazil	2014	Platelets (apheresis)	Aplastic anemia and neutropenic colitis.	Yes	Yes	No	Serology IgG/IgM RT-PCR	1	N.P.	Yes	No	−	Confirmed		
Sabino 2015 [36]	Original article	Brazil	2012	Platelets	Not specified	−	−	−	Serology IgG/IgM NAT	4	−	−	−	−	Probable	Different donors	
				FFP	Not specified	−	−	−			−	−	−	−	Probable		
				RBC	Not specified	−	−	−			−	−	−	−	Probable		
				RBC	Not specified	−	−	−			−	−	−	−	Probable		
				Platelets	Not specified	−	−	−			−	−	−	−	Probable		
				FFP (2 units)	Not specified	−	−	−			−	−	−	−	Possible		
Alves 2016 [37]	Conference abstract	Brazil	Not specified, 2015–2016?	Platelets	Lymphoblastic lymphoma—autologus H.S.C.T.	Yes	Yes	−	Serology IgM RT-PCR Sequencing NS1 Phylogenetic analysis	−	N.P.	Yes	−	−	Confirmed		
Karim 2016 [38]	Original article	Pakistan	2015	Platelets	Coronary artery bypass grafting	No	Yes	−	Serology IgM RT-PCR	−	N.P.	Yes	Yes	Yes	Possible	Same donor RBC recipient was not tested for dengue virus	
				FFP	Coronary artery bypass surgery	No	Yes	−			N.P.	No	Yes	Yes	Possible		
Matos 2016 [39]	Original article	U.S.A.	2010	RBC	Lung cancer, coronary heart disease	Yes	Yes	No	Serology IgM/IgG RT-PCR	4	−	−	−	−	Probable	Different donors	
			2011	RBC	Pancreatic cancer, DM, dementia.	Yes	Yes	No	Serology IgM	−	−	−	−	−	Possible		
Santos 2020 [40]	Case report	Brazil	2019	RBC	Sickle cell disease on chronic transfusion therapy	Yes	Yes	No	RT-PCR In house sequencing Phylogenetic analysis	2	−	−	−	−	Confirmed		

CKD: chronic kidney disease, DM: diabetes mellitus, FFP: fresh frozen plasma, HI: hemagglutination inhibition assay, HSCT: hematopoietic stem cell transplantation, IC: immunocompromised, PL: plasma, NAT: nucleic acid testing, NP: not probable, RBC: red blood cells, RT-PCR: reverse transcription polymerase chain reaction, SOT: solid organ transplantation.

− Data not available

* PL not transfused was positive

**Table 3:** Transfusion-transmitted West Nile virus cases.

Publication	Type of publication	Country	Year	Blood product	Recipient	IC.	Morbility	Mortality	Diagnostic method	Alternative explanation				Plausibility	Addtitional data	Other non-vector, non-blood transfusion related transmission
										*Exposure to Vector*	*vProlonged hospital stay*	Other infected recipients	Non-vector vias rejected			
Pealer 2003 [46]	Original article	U.S.A.	2002	RBC	Post partum	No	Yes	No	Serology IgM RT-PCR	Possible	−	−	−	Confirmed	16 implicated blood donors	S.O.T. transmission [57] Screening in blood donors [58] Vertical transmission [12]
				Platelets	Liver transplantation	Yes	Yes	No		−	−	−	−	Confirmed		
				RBC	Post partum	No	Yes	No		−	−	−	−	Confirmed		
				Platelets (2 units)	Acute myeloid leukemya	Yes	Yes	No		−	−	−	−	Confirmed		
				RBC	Lung cancer	Yes	Yes	Yes		−	−	−	−	Confirmed		
				FFP	Breast cancer	Yes	Yes	No		−	−	Yes	−	Confirmed		
				Platelets	Acute myeloid leukemya—BMT	Yes	Yes	Yes		−	−	−	−	Confirmed		
				FFP	Aneurysm repair	No	No	No		−	−	Yes	−	Confirmed		
				RBC	Acute myeloid leukemya—HSCT	Yes	No	No		−	−	Yes	−	Confirmed		
				Platelets	Rhabdomyosarcoma	Yes	Yes	No		−	−	−	−	Confirmed		
				RBC	Pneumonia, septic shock	No	No	No		−	−	Yes	−	Confirmed		
				RBC	Coronary-artery bypass grafting	No	Yes	Yes		N.P.	Yes	−	−	Confirmed		
				Platelets	Aortic-valve replacement	No	No	No		−	−	Yes	−	Confirmed		
				Platelets	Coronary-artery bypass grafting	No	Yes	No		−	−	−	−	Confirmed		
				RBC	Acute myloid leukemya—BMT	Yes	No	Yes		−	−	−	−	Confirmed		
				Platelets	Congenital bone marrow failure—HSCT	Yes	Yes	No		−	−	−	−	Confirmed		
				FFP	Motor vehicle accident	No	No	No		−	−	−	−	Confirmed		
				RBC	Lumbar laminectomy	No	Yes	No		−	−	−	−	Confirmed		
				RBC	Femur-fracture repair	No	No	No		−	−	Yes	−	Confirmed		
				RBC	Motor vehicle accident	No	No	Yes		−	−	−	−	Confirmed		
				RBC	Kidney transplantation	Yes	Yes	Yes		−	−	−	−	Confirmed		
				RBC	Gastrointestinal bleeding	No	Yes	No		−	−	−	−	Confirmed		
				RBC	Aplastic anemia	Yes	Yes	Yes		−	−	−	−	Confirmed		
Caglioti 2004 [47]	Case report	U.S.A.	2004	RBC	Above-knee amputation—DM	Yes	Yes	Yes	Serology IgM RT-PCR	Possible	No	No *	Yes	Probable		
Kightlinger 2006 [48]	Case report	U.S.A.	2006	RBC	Kidney transplantation	Yes	Yes	No	Serology IgM	N.P.	Yes	Yes	Yes	Probable	Traceback case 1 RBC	
				FFP	Kidney transplantation, DM, spinal fracture	Yes	Yes	No		−	−	Yes	−	Probable		
Montgomery 2006 [49]	Original article	U.S.A.	2003	RBC	Coronary artery bypass graft surgery	No	Yes	No	Serology IgM+ PRNT RT-PCR	N.P.	Yes	No	Yes	Confirmed	Different donors MP-NAT nonreactive.	
				RBC	Myelodysplastic syndrome	Yes	Yes	Yes		−	−	No *	Yes	Probable		
				RBC	Motor vehicle accident	No	Yes	No		−	−	No *	Yes	Probable		
				RBC	Coronary bypass, aortic valve replacement	No	Yes	No		−	No	No *	Yes	Probable		
				RBC	Coronary artery bypass graft surgery	No	No	No		−	−	No *	Yes	Probable		
				Not specified	Coronary artery bypass graft surgery	No	Yes	No		−	−	−	Yes	Probable		
Stanley 2009 [50]	Case report	U.S.A.	2008	FFP	Heart transplant	Yes	Yes	No	Serology IgG+ PRNT	N.P.	−	Yes	Yes	Probable	Traceback case 1 FFP	
				RBC	Atrial fibrillation, anemia	No	No	No		−	−	Yes	−	Probable		
Meny 2011 [51]	Letter to the editor	U.S.A.	2010	Granulocytes (apheresis)	B-lymphoblatic leukemia	Yes	Yes	Yes	Serology IgG/IgM NAT	−	Yes	No	−	−	NAT results after transfusion	
Politis 2011 [52]	Conference abstract	Greece	2010	Not specified (RBC?)	Thalassaemia	No	Yes	−	Serology IgG/IgM PCR	−	−	−	−	Confirmed	Different donors. One aditional inconclusive case	
				Not specified (RBC?)	Thalassaemia	No	Yes	−		−	−	−	−	Confirmed		
Kelly 2013 [53]	Case report	U.S.A.	2012	Platelets (apheresis)	Non-Hodgkin’s lymphoma—HSCT	Yes	Yes	Yes	Serology IgM PRNT RT-PCR	N.P.	Yes	No *	Yes	Probable	MP-NAT nonreactive.	
Pervanidou 2014 [54]	Original article	Greece	2012	Not specified	Not specified	−	Yes	No	Not specified	N.P.	−	Yes	−	−	Same donor	
				Not specified	Not specified	−	No	No	Not specified	−	−	Yes	−			
Groves 2017 [55]	Original article	U.S.A.	2016	FFP	Aortic valve replacement and coronary bypass surgery	No	Yes	Yes	Serology IgG/IgM	N.P.	No	−	Yes	Probable	MP-NAT nonreactive No data available about RBC recipient.	
Hayes 2018 [56]	Conference abstract	U.S.A.	2017	Platelets (apheresis)	Heart transplant	Yes	Yes	Yes	Serology IgG/IgM PCR	−	No	No	Yes	Probable	ID-NAT negative	

BMT: bone marrow transplantation, CKD: chronic kidney disease, DM: diabetes mellitus, FFP: fresh frozen plasma, HSCT: hematopoietic stem cell transplantation, MP: minipool, NAT: nucleic acid testing, NP: not probable, PL: plasma, PRNT: plaque reduction neutralization test, RBC: red blood cells, RT-PCR: reverse transcription polymerase chain reaction, SOT: solid organ transplantation

− Data not available

* PL not transfused was positive

### Arboviruses with a potential threat to transfusion safety

In addition to the 10 arboviruses involved in reported cases of transfusion-transmitted infections, 18 additional arboviruses were identified with a potential threat to transfusion safety by means of organ and/or hematopoietic stem cell transplantation, mother-to-child transmission, direct blood contact and/or prevalence in blood donors. The characteristics of published cases on these arboviruses are listed in Table 4. Reference studies on these other arboviruses describing the risk of direct transmission, are depicted in Table 5.

**Table 4:** Arboviruses that could pose a threat to transfusion safety due to direct non-vector related transmission mechanisms.

Source of threat to transfusion safety	Arboviruses
Transmission through organ—and/or hematopoietic stem cell transplantation	Dengue virus, eastern equine encephalitis virus, heartland virus, Jamestown Canyon virus, tick-borne encephalitis virus, and West Nile virus
Mother-to-child transmission	Colorado tick fever virus, Japanese encephalitis virus, Ross River virus, yellow fever virus, Zika virus, dengue virus, West Nile virus, chikungunya virus, Crimean-Congo hemorrhagic virus, La Crosse virus, O’nyong’nyong virus, Rift Valley fever virus, Sindbis virus, Venezuelan equine encephalitis virus and Western equine encephalitis virus.
Prevalence in blood donors	Japanese encephalitis virus, Ross River virus, St. Louis encephalitis virus, tick-borne encephalitis virus, yellow fever virus, Zika virus, dengue virus, West Nile virus, Barmah Forest Virus, chikungunya virus, Crimean-Congo hemorrhagic virus, eastern equine encephalitis virus, Heartland virus, Jamestown Canyon virus, Mayaro virus, Murray Valley encephalitis virus, O’nyong’nyong virus, Rift Valley fever virus, Huaiyangshan banyangvirus (Severe fever with thrombocytopenia syndrome Virus), Sindbis virus, Tahyna virus, Toscana virus, and Piry vesiculovirus.
Other direct routes of transmission	*Blood contact*: tick-borne encephalitis virus, dengue virus, chikungunya virus, Crimean-Congo haemorrhagic virus, La Crosse virus, Zika virus

**Table 5:** Reference studies on arboviruses that could be a threat to transfusion safety.

Virus	Publication	Type of publication	Threat to transfusion safety–Direct transmission (non-vector related transmission)
**Barmah Forest Virus**	Faddy 2014 [59]	Short communication	**Screening in blood donors:** 5.791 samples screened in 2011 by ELISA IgM: 68 positives (1,17%).
**Chikungunya virus**	Parola 2006 [60]	Original article	**Blood contact:** A 60-year-old female nurse developed symptom 3 days after contact with a patient with dengue. Interview and examination did not show recent travel abroad, mosquito bite, accidental skin puncture during blood sampling, skin lesion, or eczema. She noted direct contact with patient blood during hemostasis.
	Simmons 2016 [61]	Original article	**Screening in blood donors:** 3.007 samples screened by NAT: 56 (1.86%) were confirmed positive.
	Villamil-Gómez 2015 [62]	Original article	**Vertical transmission:** From September 2014 to February 2015, 7 pregnant women with serological and reverse transcription-polymerase chain reaction-positive test for CHIK delivered 8 infants with CHIK.
	Couderc 2012 [63]	Original article	**Other:** 4 infected corneas from apparently uninfected donors.
**Crimean-Congo haemorrhagic virus**	Tsergouli 2019[64]	Original article	**Blood contact:** Between 1953–2016: 158 cases of nosocomial transmission (2 needle-stick injury or abrasion).
	Charlier 2017 [12]	Original article	**Vertical transmission:** Transmission documented: miscarriages, stillbirths with maternal death and 1 case of documented fatal neonatal infection.
	Barabás 2018 [65]	Conference abstract	**Screening in blood donors:** Screening of 1885 blood donors with an IFA in-house method, 10 were positive (0.53%).
**Eastern equine encephalitis virus**	Leiby 2014 [66]	Conference abstract	**Screening in blood donors:** 1.314 samples have been screened by PRNT: 2 (0.15%) were confirmed positive.
	Pouch 2019 [67]	Original article	**Solid organ transplantation:** In 2017, 3 solid organ (heart, liver and lung) transplant recipients from a common donor developed encephalitis by Eastern equine encephalitis virus (EEEV).
**Heartland virus**	Hevey 2019 [68]	Case report	**Solid organ transplantation:** Heartland virus infection in a heart transplant recipient from the Heartland (U.S.A.).
	Lindsey 2019 [69]	Orginal article	**Screening in blood donors:** Of the 487 blood donors tested, 12 were positives, and 7 of those were confirmed for Heartland virus neutralizing antibodies by PRNT.
**Jamestown Canyong virus**	Askar 2019 [70]	Case report	**Solid organ transplantation:** Jamestown Canyon virus encephalitis in a heart transplant patient.
	Mayo 2001 [71]	Letter to the Editor	**Screening in blood donors:** Of the 1,086 sera collected from blood donors in 1990, 164 (15%) were positive by IFA.
**La Crosse virus**	R. Bowen 2013 [72]	Conference abstract	**Blood contact:** During the 2011 outbreak in China it was discovered that the virus was able to be transmitted person to person through blood contact, indicating the possibility this virus could be transmitted through a tainted blood product.
	Charlier 2017 [12]	Original article	**Vertical transmission:** Transmission documented: 1 asymptomatic mother-to-child transmission documented serologically in cord serum, after a maternal infection.
**Mayaro virus**	Romeiro 2020 [73]	Original article	**Screening in blood donors:** 5608 blood donor samples were tested. IgM and IgG antibodies to MAYV were detected in 36 and 11 samples, respectively.
**Murray valley encephalitis virus**	Williams 2013 [74]	Original article	**Screening in blood donors:** 592 samples from blood donors from the Murray Valley region, 6 positives for IgG antibodies (1%; 95% CI: 0.2–1.8%)
**O’nyong’nyong virus**	Clements 2019 [75]	Original article	**Screening in blood donors:** 24 of 552 CHIKV IgG-positive samples (4.4%) were tested for their ability to neutralize CHIKV and other related alphaviruses. 23 of the samples showed higher titers against ONNV than CHIKV.
	Charlier 2017 [12]	Original article	**Vertical transmission:** Transmission uncertain; 2 miscarriages reported.
**Rift Valley fever virus**	Azhar 2010 [76]	Original article	**Screening in blood donors:** 2 of 1260 (0.16%) blood donors were RVFV IgG positive.
	Charlier 2017 [12]	Original article	**Vertical transmission:** Transmission documented; increased risk of miscarriage. Two symptomatic cases of mother-to-child transmission.
**SFTS Virus**	Zeng 2015 [77]	Original article	**Screening in blood donors:** 14,764 donor samples were tested for anti-SFTSV IgG antibodies, 86 of which were repeat-reactive and defined as seropositive.
**Sindbis virus**	Jöst 2011 [78]	Letter to the Editor	**Screening in blood donors:** 389 investigated blood donor samples were tested for SINV-specific-IgG antibodies, 4 were positive.
	Charlier 2017 [12]	Original article	**Vertical transmission:** Transmission uncertain; two stillbirths reported.
**Tahyna virus**	Sonnleitner 2014 [79]	Original article	**Screening in blood donors:** IgG antibodies were determined in sera of 1630 blood donors. 10 (0.6%) reacted positive by TAHV IFA, 5 of which (0.3%) were confirmed by neutralization test.
**Toscana virus**	Brisbarre 2011 [80]	Letter to the Editor	**Screening in blood donors:** 84 (11.5%) of 729 plasma samples were positive for IgG. 24 (3.3%) were positive for IgM, and 5 (0.7%) were positive for IgG and IgM.
**Venezuelan equine encephalitis virus**	Charlier 2017 [12]	Original article	**Vertical transmission:** virus documented in the brains of ten aborted fetuses; developmental brain lesions in infants borne by infected mothers.
**Vesiculovirus Piry**	Tavares-Neto 1990 [81]	Original article	**Screening in blood donors:** frequency of neutralizing antibodies 8% (13/162).
**Western equine encephalitis virus**	Charlier 2017 [12]	Original article	**Vertical transmission:** three cases with severe encephalitis.

IFA: indirect fluorescent antibody, NAT: nucleic acid testing, PRNT: plaque reduction neutralization test, SFTS Virus: Severe fever with thrombocytopenia syndrome virus (Huaiyangshan banyangvirus).

## Discussion

Until the year 2000, transfusion transmission of arboviruses was almost anecdotal, with only one isolated case of transmission of Colorado tick fever virus and two cases of tick-borne encephalitis virus. However, after the West Nile virus outbreak in the USA in 2002, the number of cases increased considerably [46]. Since then, more than 70 cases of arbovirus transmission have been published, including several cases of emerging arboviruses: Japanese encephalitis virus, Ross River virus, St. Louis encephalitis virus, Powassan virus, and Zika virus [13,15,17,19,26,27].

This study confirms the possibility that arboviruses can be transmitted through transfusion of blood and blood components. A total of 74 reported cases of suspected transfusion-transmitted infections from 10 arboviruses were identified. Infections were reported to be symptomatic in 63.5% and fatal in 18.9% of the reported cases. This transmission route therefore poses a considerable threat to transfusion safety, reportedly causing significant morbidity and mortality and an increased health spending. As more than 50% of affected patients were classified as immunocompromised, transmission of arboviruses through blood or blood components can additionally be considered a threat to a particularly vulnerable section of the population. Analysis of reported cases revealed that transfusion transmission occurred through various blood products. It is observed that transmission through platelet transfusion was found to be higher in cases of dengue virus transmission, compared to other arboviral transmission cases, including the West Nile virus cases. This finding could be related to signs of tropism of the dengue virus against platelets or at least some of their components, such as glycoprotein Ib (CD42b) [82].

In addition to the published cases of transfusion transmission of arboviruses, our review identified 18 additional arboviruses with potential threat to transfusion safety through solid organ transplantation, mother-to-child transmission, blood contact or prevalence in blood donors. The results of this review further confirm identification of arboviruses with a high-risk of future transfusion transmission, as they were reported to be involved in direct non-vector related transmission and prevalent among blood donors. These are: Chikungunya virus, Crimean-Congo haemorrhagic virus, Eastern equine encephalitis virus, Heartland virus and Jamestown Canyong virus.

Based on the results of this review, the reported rapid growth of arboviral cases in recent years can also be confirmed and seems to correspond to the expanding endemicity of arboviruses globally. According to literature, the increased frequency of outbreaks can be explained by multiple environmental and anthropogenic factors, including increased travel and trade, population growth, changing land use patterns, and the urbanization of rural areas [83].

These facts come on top of a growing list of emerging infectious diseases. It is estimated that from 1940 to 2004, 5.3 new viruses were discovered each year, of which at least two-thirds were zoonoses [84]. In consonance with their emerging nature, there is very little knowledge on some of these new arboviruses, such as the SFTS virus, which has already caused multiple deaths in South Korea [85].

To improve transfusion safety, blood banks have taken a number of measures. One common measure is the exclusion of donors presenting symptoms. This is a measure with limited effectiveness given the high rate of asymptomatic infections. Another measure taken is the cancellation of blood donations in affected regions, which is at times difficult to apply, if the epidemic outbreak is extensive and no contingency plans are in place to guarantee the supply of blood and blood components from other geographical regions. So far, the most useful measure is the application of nucleic acid testing (NAT) in donor screening. One example illustrating this success comes from the significant reduction in transmission of West Nile virus in the USA, after the implementation of NAT screening in 2003. However, NAT tests are not infallible: for instance, one case of West Nile virus transmission was reported in a donor with very low viremia who had undergone NAT screening, using a simple sample [56]. Another compromising aspect is the expense: the implementation of NAT testing for West Nile virus in the USA, had an estimated cost of USD 483,000–1,300,000 per quality-adjusted life year (QALY), clearly exceeding the widely accepted threshold of USD 50,000–100,000 per QALY [3,86]. Furthermore, implementing NAT tests requires human and logistical resources that are not available in all settings. As an alternative, techniques for the inactivation of pathogens such as methylene blue and visible light, could also be applied where available, as these methods were proven effective against several arboviruses [87,88]. In general, and despite limitations, the best probable strategy is probably to screen using NAT techniques and apply pathogen inactivation techniques to as many blood components as resources allow.

In addition, blood transfusion centers should develop robust hemovigilance systems with the ability to trace blood products from infected donors, so that these can be destroyed prior to use or, in case these have already been transfused, the physician responsible for clinical management of the recipient, can be informed in a timely fashion.

The risk of transfusion-transmitted arbovirus infections is of particular concern in low-income countries, usually in endemic areas or areas where extensive epidemic outbreaks occur. Particularly since blood bank systems are usually not based on voluntary donations, but rather on replacement donations. In addition, testing for infectious agents is not optimal, due to technical and resource constraints or a lack of qualified personnel, often relying on rapid diagnostic tests, known to have a poor sensitivity [89,90].

One limitation of this systematic review is the inherent difficulty of confirming the transmission of the arbovirus through the transfusion of blood components. Unequivocally concluding that an infection was transmitted through transfusion would require demonstrating that the infectious agent was found in samples from the donor or in residual blood components from the donation, that the recipient was not infected before receiving the transfusion, and that the virus was not transmitted through the bite of a vector. This is especially complicated in endemic areas, where the population is exposed to vector bites (mainly mosquitoes), with the possible exception of patients admitted to specific units such as: units specialized in transplantation and intensive care units. However, even in these “mosquito free” units, patients could have been infected before hospital admission. Phylogenetic analysis may be relevant to exclude the transfusion hypothesis, but if the donor and recipient reside in the same geographic area, the same genetic sequence may not be enough to confirm this exclusion [91]. Therefore, definitively classifying a case as transfusion transmission is often unfeasible.

Similarly, a significant number of transfusion-transmitted arbovirus infections are likely to go undetected. If, despite the transmission, the recipient does not present symptoms, or if the transmission occurs in a geographical region during an epidemic outbreak, where transmission through the vector cannot be ruled out, transfusion transmission may go unnoticed.

Another limitation of the study is that conclusions of authors of the reviewed records on the plausibility of transfusion transmission and mortality as a consequence of the arboviral infection, were copied with limited information available to confirm these independently. In addition, each author may have applied different criteria in establishing the plausibility of arboviral transmission through the transfusion of blood or blood components and mortality as a possible outcome.

In conclusion, this review updated, characterized and classified cases of arbovirus transmission through transfusion. In addition, a significant number of arboviruses were identified with a potential threat to future transfusion safety. In recent years a global increase was seen in the number of epidemic outbreaks of various arboviruses. In the coming years, it is expected that this number will continue to increase. For this reason, it is important that each system or organization responsible for transfusion safety has adequate surveillance systems, contingency plans, and protocols in place to guarantee transfusion safety. The different regulatory agencies, public health bodies, medical communities, and the medical industry should collaborate closely and coordinate efforts to provide a rapid and effective response to any threat to transfusion safety.

## Supporting information

S1 Table PICOT research question. (DOCX)

S1 PRISMA Checklist (DOCX)

S1 Text Search strategy. (DOCX)

S1 Flow Chart Flow chart on selection of case studies of transfusion-transmitted arboviruses. (DOCX)

S2 Flow Chart Flow chart for the process of selecting records describing other mechanisms of direct transmission. (DOCX)
