# Supplementary material for: Transfusion-transmitted arboviruses: Update and systematic review
Source: PLoS Negl Trop Dis. 2022 Oct 6;16(10):e0010843. doi: 10.1371/journal.pntd.0010843 (PMC9578600; doi:10.1371/journal.pntd.0010843)
Supplement: S1 Table — (DOCX) [file pntd.0010843.s001.docx]

PICOT research question: What are the published cases of transfusion-transmitted arboviruses throughout the history of transfusion?

|  | |
| --- | --- |
| **PICOT** | **Description** |
| **P**roblem | Transmission of arboviruses trough blood transfusion |
| **I**ntervention | Reported and published in scientific literature |
| **C**omparison | Not applicable |
| **O**utcome | Reported and published transfusion-transmitted arboviruses cases and their epidemiological and clinical characteristics |
| **T**ime | Throughout the history of transfusion |
