# Supplementary material for: Transfusion-transmitted arboviruses: Update and systematic review
Source: PLoS Negl Trop Dis. 2022 Oct 6;16(10):e0010843. doi: 10.1371/journal.pntd.0010843 (PMC9578600; doi:10.1371/journal.pntd.0010843)
Supplement: S1 PRISMA Checklist — (DOCX) [file pntd.0010843.s002.docx]

| **Section/topic** | **#** | **Checklist item** | **Reported on page #** |
| --- | --- | --- | --- |
| **TITLE** | | |  |
| Title | 1 | Identify the report as a systematic review, meta-analysis, or both. | Page 1. Transfusion-transmitted arboviruses: update and systematic review. |
| **ABSTRACT** | | |  |
| Structured summary | 2 | Provide a structured summary including, as applicable: background; objectives; data sources; study eligibility criteria, participants, and interventions; study appraisal and synthesis methods; results; limitations; conclusions and implications of key findings; systematic review registration number. | Page 2. Structured abstract in **Background**, **Material and methods**, **Results** and **Discussion**. |
| **INTRODUCTION** | | |  |
| Rationale | 3 | Describe the rationale for the review in the context of what is already known. | Page 5. “…major global public health entities have expressed the need for comprehensive knowledge of the epidemiological characteristics of the various emerging transfusion-transmitted infections and to evaluate their potential impact on donor selection criteria and the supply of blood and blood products.” |
| Objectives | 4 | Provide an explicit statement of questions being addressed with reference to participants, interventions, comparisons, outcomes, and study design (PICOS). | Page 5-6. “The main objective of this study is, therefore, to review the published cases of arbovirus transmission through the transfusion of blood or blood components throughout the history of transfusion and to study their main clinical and epidemiological characteristics. For the research question in PICOT format, please see supplementary file (S1). The secondary objective is to, additionally, identify”  Supplemental File S1 |
| **METHODS** | | |  |
| Protocol and registration | 5 | Indicate if a review protocol exists, if and where it can be accessed (e.g., Web address), and, if available, provide registration information including registration number. | Page 7. The protocol for this systematic review was published and registered in PROSPERO (www.crd.york.ac.uk/prospero, CRD42021270355). |
| Eligibility criteria | 6 | Specify study characteristics (e.g., PICOS, length of follow-up) and report characteristics (e.g., years considered, language, publication status) used as criteria for eligibility, giving rationale. | Page 8-9. **Study selection**  “In answer to the primary objective, selected publications were those in any language describing cases of transfusion-transmitted arboviruses transmitted through transfusion of blood or blood components. A wide variety of scientific studies (original articles, reviews, brief reports, case reports, letters to the editor, conference papers and others) were included reporting cases of arboviruses transmitted through transfusion of blood or blood components. Publications that collected cases of transmission in non-humans were excluded, as were publications that did not detail the specific type of arbovirus transmitted through the transfusion of blood or blood components.” |
| Information sources | 7 | Describe all information sources (e.g., databases with dates of coverage, contact with study authors to identify additional studies) in the search and date last searched. | Page 7. **Source of data collection**  “…Systematic literature searches were conducted in MEDLINE, Embase, Scopus using the server of University Miguel Hernández server.” |
| Search | 8 | Present full electronic search strategy for at least one database, including any limits used, such that it could be repeated. | Pages 7-8. **Search strategy**  “…The complete search strategy is described in more detail in the study protocol and Supplementary File (S3). Initially, the authors have performed searches with free text terms in all three bibliographic databases using the term "transfusion" in combination with the name of the arboviruses being reviewed. In MEDLINE, Medical Subject Headings (MeSH) such as “Blood Transfusion”[Mesh] OR “Transfusion Reaction”[Mesh] also ...”  Supplemental File S3. |
| Study selection | 9 | State the process for selecting studies (i.e., screening, eligibility, included in systematic review, and, if applicable, included in the meta-analysis). | Page 8-9. **Study selection**  “…Search records were independently evaluated by two review authors for inclusion in the publication overview. Any discrepancies were resolved by consensus. Inter-observer agreement was measured by determining the kappa index...”  Figure 1. PRISMA flow chart  Supplemental File S4.  Supplemental File S5. |
| Data collection process | 10 | Describe method of data extraction from reports (e.g., piloted forms, independently, in duplicate) and any processes for obtaining and confirming data from investigators. | Page 9-10. **Data extraction**  “…All data of cases of transfusion-transmitted arboviruses were extracted from the selected publications and directly entered into a Microsoft Excel database by a single review author. Data quality control was consequently done by a second review author...” |
| Data items | 11 | List and define all variables for which data were sought (e.g., PICOS, funding sources) and any assumptions and simplifications made. | Page 9. “…If a publication included more than one case of transfusion transmission, each case was recorded and entered separately in the database. Basic epidemiological and clinical data of the reported cases of transfusion transmission were then analyzed: type of blood component implicated, immunosuppression and clinical presentation in the recipient, fatality of the outcome following transmission as well as the plausibility of the respective transmission route...”  Supplemental File S1 |
| Risk of bias in individual studies | 12 | Describe methods used for assessing risk of bias of individual studies (including specification of whether this was done at the study or outcome level), and how this information is to be used in any data synthesis. | Page 10. “Due to the mainly descriptive objective of this our study and the type of publications included (case reports, letters to the editor), no standard quality control tool, such as ROBINS-I or STROBE checklist, was applied for assessing risk of bias in non-randomized studies...” |
| Summary measures | 13 | State the principal summary measures (e.g., risk ratio, difference in means). | Pages 10. “Results were expressed as proportion with a 95% confidence interval (95% CI). The proportion was calculated as the ratio of the reported parameter under study divided by the total number of reported cases of transfusion transmission (Example: number of cases of transfusion-transmitted arboviruses related with the transfusion of red blood cells / total number of cases of transfusion-transmitted arboviruses x 100). The 95% CIs were for proportions (p±Zα/2√(p(1-p)/n)).” |
| Synthesis of results | 14 | Describe the methods of handling data and combining results of studies, if done, including measures of consistency (e.g., I^2^) for each meta-analysis. | Not applicable |

Page 1 of 2

| **Section/topic** | **#** | **Checklist item** | **Reported on page #** |
| --- | --- | --- | --- |
| Risk of bias across studies | 15 | Specify any assessment of risk of bias that may affect the cumulative evidence (e.g., publication bias, selective reporting within studies). | Not applicable. |
| Additional analyses | 16 | Describe methods of additional analyses (e.g., sensitivity or subgroup analyses, meta-regression), if done, indicating which were pre-specified. | Page 7. “Finally, a descriptive analysis was performed of arboviruses that can be considered a potential threat to transfusion safety, based on published reports of any non-vector, non-blood related direct route of transmission or by their prevalence in blood donors, regardless of reported evidence of transfusion transmission”. |
| **RESULTS** | | |  |
| Study selection | 17 | Give numbers of studies screened, assessed for eligibility, and included in the review, with reasons for exclusions at each stage, ideally with a flow diagram. | Page 11. “The search strategy yielded a total of 5158 records. After eliminating duplicates, the authors screened the title and abstract of 2918 records and retrieved the full text of 199. The authors selected any records that suggest human transmission of arboviruses through blood transfusion or studies that …”  Figure 1. PRISMA flow chart on selection of included studies covered by the literature review |
| Study characteristics | 18 | For each study, present characteristics for which data were extracted (e.g., study size, PICOS, follow-up period) and provide the citations. | Tables 1, 2 and 3. |
| Risk of bias within studies | 19 | Present data on risk of bias of each study and, if available, any outcome level assessment (see item 12). | Not applicable. |
| Results of individual studies | 20 | For all outcomes considered (benefits or harms), present, for each study: (a) simple summary data for each intervention group (b) effect estimates and confidence intervals, ideally with a forest plot. | Tables 1, 2 and 3. |
| Synthesis of results | 21 | Present results of each meta-analysis done, including confidence intervals and measures of consistency. | Pages 12-16. … “The USA was the country with the most reported cases of transfusion transmission (N=42, 62%.2%, 95% CI: 51.1-73,2%). The main type of blood component implicated was red blood cells (N=35, 47.3%; 95% CI: 35.9-58.7%) ...” |
| Risk of bias across studies | 22 | Present results of any assessment of risk of bias across studies (see Item 15). | Not applicable. |
| Additional analysis | 23 | Give results of additional analyses, if done (e.g., sensitivity or subgroup analyses, meta-regression [see Item 16]). | Page 21. “In addition to the 10 arboviruses involved in reported cases of transfusion transmitted infections, 18 additional arboviruses were identified with a potential threat to transfusion safety by means of organ and/or hematopoietic stem cell transplantation, mother-to-child transmission, direct blood contact and/or prevalence in blood donors…”  Tables 4 and 5. |
| **DISCUSSION** | | |  |
| Summary of evidence | 24 | Summarize the main findings including the strength of evidence for each main outcome; consider their relevance to key groups (e.g., healthcare providers, users, and policy makers). | Page 23. “This study confirms the possibility that arboviruses can be transmitted through transfusion of blood and blood components. A total of 74 reported cases of suspected transfusion-transmitted infections from 10 arboviruses were identified. Infections were reported to be symptomatic in 63.5% and fatal in 18.9% of the reported cases. This transmission route therefore poses a considerable threat to transfusion safety ...” |
| Limitations | 25 | Discuss limitations at study and outcome level (e.g., risk of bias), and at review-level (e.g., incomplete retrieval of identified research, reporting bias). | Page 25. “One limitation of this systematic review is the inherent difficulty of confirming the transmission of the arbovirus through the transfusion of blood components. Unequivocally concluding that an infection was transmitted through transfusion would require demonstrating that the infectious agent was found in samples from the donor or in residual blood components from the donation, that the recipient was not infected before receiving the transfusion, and that the virus was not transmitted through the bite of a vector. This is especially complicated in endemic areas, where the population is exposed to vector bites (mainly mosquitoes), with the possible exception of …”. |
| Conclusions | 26 | Provide a general interpretation of the results in the context of other evidence, and implications for future research. | Page 26. “In conclusion, this review updated, characterized and classified cases of arbovirus transmission through transfusion. In addition, a significant number of arboviruses were identified with a potential threat to future transfusion safety. In recent years a global increase was seen in the number of epidemic outbreaks of various arboviruses. In the coming years, it is expected that this number will continue to increase...” |
| **FUNDING** | | |  |
| Funding | 27 | Describe sources of funding for the systematic review and other support (e.g., supply of data); role of funders for the systematic review. | Page 27. “This research did not receive any specific grant from funding agencies in the public, commercial, or not-for-profit sectors”. |

*From:*  Moher D, Liberati A, Tetzlaff J, Altman DG, The PRISMA Group (2009). Preferred Reporting Items for Systematic Reviews and Meta-Analyses: The PRISMA Statement. PLoS Med 6(7): e1000097. doi:10.1371/journal.pmed1000097
