# Supplementary material for: Transfusion-transmitted arboviruses: Update and systematic review
Source: PLoS Negl Trop Dis. 2022 Oct 6;16(10):e0010843. doi: 10.1371/journal.pntd.0010843 (PMC9578600; doi:10.1371/journal.pntd.0010843)
Supplement: S1 Text — (DOCX) [file pntd.0010843.s003.docx]

**Transfusion-transmitted arboviruses: update and systematic review**

*S1 Text*

First search strategy

**P** = Pubmed, **E** = Embase, **S** = Scopus

**Bunyamwera virus**

**P** transfusion AND Bunyamwera virus

**P** ("Blood Transfusion"[Mesh] OR "Transfusion Reaction"[Mesh]) AND "Bunyamwera virus"[Mesh]

**E** ('transfusion'/exp OR transfusion) AND ('bunyamwera virus'/exp OR 'bunyamwera virus')

**S** TITLE-ABS-KEY ( transfusion AND “bunyamwera virus” )

**Crimean Congo hemorrhagic fever virus**

**P** transfusion AND crimean congo hemorrhagic fever virus

**P** ("Blood Transfusion"[Mesh] OR "Transfusion Reaction"[Mesh]) AND "Hemorrhagic Fever Virus, Crimean-Congo"[Majr]

**E** ('transfusion'/exp OR transfusion) AND ('crimean-congo hemorrhagic fever virus'/exp OR 'crimean-congo hemorrhagic fever virus')

**S** TITLE-ABS-KEY ( transfusion AND "crimean congo hemorrhagic fever virus" )

**Heartland virus Blood donors**

**P** transfusion AND Heartland virus

**P** "Transfusion Reaction"[Mesh] OR "Blood Transfusion"[Mesh] AND Heartland virus

**E** ('transfusion'/exp OR transfusion) AND ('heartland virus'/exp OR 'heartland virus')

**S** TITLE-ABS-KEY ( transfusion AND “heartland virus” )

**Huaiyangshan banyangvirus (severe fever with thrombocytopenia syndrome virus)**

**P** transfusión AND banyangvirus

**P** "Blood Transfusion"[Mesh] OR "Transfusion Reaction"[Mesh] AND banyangvirus

**E** ('transfusion'/exp OR transfusion) AND banyangvirus

**S** TITLE-ABS-KEY ( transfusion  AND  banyangvirus )

**Jamestown Canyon virus**

**P** transfusion AND Jamestown Canyon virus

**P** "Blood Transfusion"[Mesh] OR "Transfusion Reaction"[Mesh] AND Jamestown Canyon virus

**E** ('transfusion'/exp OR transfusion) AND ('jamestown canyon virus'/exp OR 'jamestown canyon virus')

**S** TITLE-ABS-KEY ( transfusion AND “jamestown canyon virus” )

**La Crosse virus**

**P** transfusion AND la crosse virus

**P** ("Blood Transfusion"[Mesh] OR "Transfusion Reaction"[Mesh]) AND "La Crosse virus"[Majr]

**E** ('transfusion'/exp OR transfusion) AND ('la crosse virus'/exp OR 'la crosse virus')

**S** TITLE-ABS-KEY ( transfusion AND "la crosse virus" )

**Rift Valley fever virus**

**P** transfusion AND rift valley fever virus

**P** ("Blood Transfusion"[Mesh] OR "Transfusion Reaction"[Mesh]) AND ( "Rift Valley Fever"[Mesh] OR "Rift Valley fever virus"[Mesh] )

**E** ('transfusion'/exp OR transfusion) AND ('rift valley fever virus'/exp OR 'rift valley fever virus')

**S** TITLE-ABS-KEY ( transfusion AND "rift valley fever virus" )

**California enchephalitis virus (Tahyna virus)**

**P** transfusion AND california encephalitis virus

**P** ("Blood Transfusion"[Mesh] OR "Transfusion Reaction"[Mesh]) AND ( "Encephalitis, California"[Mesh] OR "Encephalitis Virus, California"[Mesh] )

**E** ('transfusion'/exp OR transfusion) AND ('california encephalitis virus'/exp OR 'california encephalitis virus')

**S** TITLE-ABS-KEY ( transfusion AND "california enchephalitis virus" )

**Toscana virus**

**P** transfusion AND toscana virus

**P** ("Blood Transfusion"[Mesh] OR "Transfusion Reaction"[Mesh]) AND "Sandfly fever Naples virus"[Majr]

**E** ('transfusion'/exp OR transfusion) AND ('toscana virus'/exp OR 'toscana virus')

**S** TITLE-ABS-KEY ( transfusion AND "toscana virus" )

**Dengue virus**

**P** transfusion AND dengue virus

**P** ("Blood Transfusion"[Mesh] OR "Transfusion Reaction"[Mesh]) AND ( "Dengue"[Mesh] OR "Dengue Virus"[Mesh] )

**E** ('transfusion'/exp OR transfusion) AND ('dengue virus'/exp OR 'dengue virus')

**S** TITLE-ABS-KEY ( transfusion AND "dengue virus" )

**Japanese encephalitis virus**

**P** transfusion AND japanese encephalitis virus

**P** ("Blood Transfusion"[Mesh] OR "Transfusion Reaction"[Mesh]) AND "Encephalitis Virus, Japanese"[Mesh]

**E** ('transfusion'/exp OR transfusion) AND ('japanese encephalitis virus'/exp OR 'japanese encephalitis virus')

**S** TITLE-ABS-KEY ( transfusion AND "japanese encephalitis virus" )

**Kyasanur forest disease virus**

**P** transfusion AND kyasanur forest disease virus

**P** ("Blood Transfusion"[Mesh] OR "Transfusion Reaction"[Mesh]) AND "Kyasanur Forest Disease"[Mesh]

**E** ('transfusion'/exp OR transfusion) AND ('kyasanur forest disease virus'/exp OR 'kyasanur forest disease virus')

**S** TITLE-ABS-KEY ( transfusion AND "kyasanur forest disease virus" )

**Louping ill virus**

**P** transfusion AND louping ill virus

**P** ("Blood Transfusion"[Mesh] OR "Transfusion Reaction"[Mesh]) AND "Louping Ill"[Mesh]

**E** ('transfusion'/exp OR transfusion) AND ('louping ill virus'/exp OR 'louping ill virus')

**S** TITLE-ABS-KEY ( transfusion AND "louping ill virus" )

**Murray valley encephalitis virus
P** transfusion AND murray valley encephalitis virus

**P** ("Blood Transfusion"[Mesh] OR "Transfusion Reaction"[Mesh]) AND "Encephalitis Virus, Murray Valley"[Majr]

**E** ('transfusion'/exp OR transfusion) AND ('murray valley encephalitis virus'/exp OR 'murray valley encephalitis virus')

**S** TITLE-ABS-KEY ( transfusion AND "murray valley encephalitis virus” )

**Spondweni virus**

**P** transfusion AND Spondweni virus

**P** "Transfusion Reaction"[Mesh] OR "Blood Transfusion"[Mesh] AND Spondweni virus

**E** ('transfusion'/exp OR transfusion) AND ('spondweni virus'/exp OR 'spondweni virus')

**S** TITLE-ABS-KEY ( transfusion AND “spondweni virus” )

**St. Louis encephalitis virus**

**P** transfusion AND st. louis encephalitis virus

**P** ("Blood Transfusion"[Mesh] OR "Transfusion Reaction"[Mesh]) AND ( "Encephalitis, St. Louis"[Mesh] OR "Encephalitis Virus, St. Louis"[Mesh] )

**E** ('transfusion'/exp OR transfusion) AND ('st. louis encephalitis virus'/exp OR 'st. louis encephalitis virus')

**S** TITLE-ABS-KEY ( transfusion AND "st. louis encephalitis virus" )

**Tick-borne encephalitis virus**

**P** transfusion AND tick-borne encephalitis virus

**P** ("Blood Transfusion"[Mesh] OR "Transfusion Reaction"[Mesh]) AND "Encephalitis Viruses, Tick-Borne"[Mesh]

**E** ('transfusion'/exp OR transfusion) AND ('tick borne encephalitis virus'/exp OR 'tick borne encephalitis virus')

**S** TITLE-ABS-KEY ( transfusion AND "tick-borne encephalitis virus" )

**Powassan virus**

**P** transfusion AND powassan virus

**P** (blood transfusion[MeSH] OR transfusion reaction[MeSH]) AND (powassan virus[MeSH])

**E** ('transfusion'/exp OR transfusion) AND ('powassan virus'/exp OR 'powassan virus')

**S** TITLE-ABS-KEY ( transfusion AND "powassan virus" )

**West Nile virus**

**P** transfusion AND west nile virus

**P** ("Blood Transfusion"[Mesh] OR "Transfusion Reaction"[Mesh]) AND ( "West Nile virus"[Mesh] OR "West Nile Fever"[Mesh] )

**E** ('transfusion'/exp OR transfusion) AND ('west nile virus'/exp OR 'west nile virus')

**S** TITLE-ABS-KEY ( transfusion AND "west nile virus" )

**Yellow fever virus**

**P** transfusion AND yellow fever virus

**P** ("Blood Transfusion"[Mesh] OR "Transfusion Reaction"[Mesh]) AND "Yellow fever virus"[Mesh]

**E** ('transfusion'/exp OR transfusion) AND ('yellow fever virus'/exp OR 'yellow fever virus')

**S** TITLE-ABS-KEY ( transfusion AND "yellow fever virus" )

**Zika virus**

**P** transfusion AND zika virus

**P** ("Blood Transfusion"[Mesh] OR "Transfusion Reaction"[Mesh]) AND ( "Zika Virus"[Mesh] OR "Zika Virus Infection"[Mesh] )

**E** ('transfusion'/exp OR transfusion) AND ('zika virus'/exp OR 'zika virus')

**S** TITLE-ABS-KEY ( transfusion AND "zika virus" )

**Banna virus**

**P** transfusion AND Banna virus

**P** "Transfusion Reaction"[Mesh] OR "Blood Transfusion"[Mesh] AND Banna virus

**E** ('transfusion'/exp OR transfusion) AND ('banna virus'/exp OR 'banna virus')

**S** TITLE-ABS-KEY ( transfusion AND "Banna virus" )

**Bluetongue virus**

**P** transfusion AND blue tongue virus

**P** ("Blood Transfusion"[Mesh] OR "Transfusion Reaction"[Mesh]) AND "Bluetongue virus"[Majr]

**E** ('transfusion'/exp OR transfusion) AND ('bluetongue orbivirus'/exp OR 'bluetongue orbivirus')

**S** TITLE-ABS-KEY ( transfusion AND "bluetongue virus" )

**Colorado tick fever virus**

**P** transfusion AND colorado tick fever virus

**P** ("Blood Transfusion"[Mesh] OR "Transfusion Reaction"[Mesh]) AND "Colorado tick fever virus"[Majr]

**E** ('transfusion'/exp OR transfusion) AND ('colorado tick fever virus'/exp OR 'colorado tick fever virus')

**S** TITLE-ABS-KEY ( transfusion AND "colorado tick fever virus" )

**Vesicular stomatitis virus (Vesiculovirus Piry)**

**P** transfusion AND vesicular stomatitis virus

**P** ("Blood Transfusion"[Mesh] OR "Transfusion Reaction"[Mesh]) AND "Vesicular stomatitis Indiana virus"[Mesh]

**E** ('transfusion'/exp OR transfusion) AND ('vesiculovirus'/exp OR vesiculovirus)

**S** TITLE-ABS-KEY ( transfusion AND "vesicular stomatitis virus" )

**Barmah forest virus**

**P** transfusion AND Barmah forest virus

**P** ("Blood Transfusion"[Mesh] OR "Transfusion Reaction"[Mesh]) AND Barmah forest virus

**E** ('barmah forest virus'/exp OR 'Barmah forest virus') AND ('transfusion'/exp OR transfusion)

**S** TITLE-ABS-KEY ( transfusion AND "Barmah forest virus" )

**Chikungunya virus**

**P** transfusion AND chikungunya virus

**P** ("Blood Transfusion"[Mesh] OR "Transfusion Reaction"[Mesh]) AND ( "Chikungunya Fever"[Mesh] OR "Chikungunya virus"[Mesh] )

**E** ('transfusion'/exp OR transfusion) AND ('chikungunya virus'/exp OR 'chikungunya virus')

**S** TITLE-ABS-KEY ( transfusion AND "chikungunya virus" )

**Eastern equine encephalitis virus**

**P** Transfusion AND Eastern equine encephalitis virus

**P** ("Transfusion Reaction"[Mesh] OR "Blood Transfusion"[Mesh]) AND "Encephalitis Virus, Eastern Equine"[Mesh]

**E** ('transfusion'/exp OR transfusion) AND ('eastern equine encephalitis virus'/exp OR 'eastern equine encephalitis virus')

**S** TITLE-ABS-KEY ( transfusion AND "Eastern equine encephalitis virus" )

**Mayaro virus**

**P** transfusion AND mayaro virus

**P** "Blood Transfusion"[Mesh] OR "Transfusion Reaction"[Mesh] AND mayaro virus

**E** ('transfusion'/exp OR transfusion) AND ('mayaro virus'/exp OR 'mayaro virus')

**S** TITLE-ABS-KEY ( transfusion AND "mayaro virus" )

**O’nyong o’nyong virus**

**P** transfusion AND o'nyong'nyong

**P** ("Blood Transfusion"[Mesh] OR "Transfusion Reaction"[Mesh]) AND "O'nyong-nyong Virus"[Majr]

**E** ('transfusion'/exp OR transfusion) AND ('o nyong nyong virus'/exp OR 'o nyong nyong virus')

**S** TITLE-ABS-KEY ( transfusion AND "o'nyong o'nyong virus" )

**Ross river virus**

**P** transfusion AND ross river virus

**P** ("Blood Transfusion"[Mesh] OR "Transfusion Reaction"[Mesh]) AND "Ross River virus"[Mesh]

**E** ('transfusion'/exp OR transfusion) AND ('ross river virus'/exp OR 'ross river virus')

**S** TITLE-ABS-KEY ( transfusion AND "ross river virus" )

**Sindbis virus**

**P** transfusion AND sindbis virus

**P** ("Blood Transfusion"[Mesh] OR "Transfusion Reaction"[Mesh]) AND "Sindbis Virus"[Mesh]

**E** ('transfusion'/exp OR transfusion) AND ('sindbis virus'/exp OR 'sindbis virus')

**S** TITLE-ABS-KEY ( transfusion AND "sindbis virus" )

**Venezuelan equine encephalitis virus**

**P** transfusion AND venezuelan equine encephalitis virus

**P** ("Blood Transfusion"[Mesh] OR "Transfusion Reaction"[Mesh]) AND "Encephalitis Virus, Venezuelan Equine"[Majr]

**E** ('transfusion'/exp OR transfusion) AND ('venezuelan equine encephalitis virus'/exp OR 'venezuelan equine encephalitis virus')

**S** TITLE-ABS-KEY ( transfusion AND "venezuelan equine encephalitis virus" )

**Western equine encephalitis virus**

**P** transfusion AND western equine encephalitis virus

**P** ("Blood Transfusion"[Mesh] OR "Transfusion Reaction"[Mesh]) AND "Encephalitis Virus, Western Equine"[Majr]

**E** ('transfusion'/exp OR transfusion) AND ('western equine encephalitis virus'/exp OR 'western equine encephalitis virus')

**S** TITLE-ABS-KEY ( transfusion AND "western equine encephalitis virus" )

Second search strategy

**P** = Pubmed, **E** = Embase,

**Bunyamwera virus**

**P** Transplantation AND Bunyamwera virus

**P** Vertical transmission AND Bunyamwera virus

**P** Blood donors AND Bunyamwera virus

**E** ('transplantation'/exp OR transplantation) AND ('bunyamwera virus'/exp OR 'bunyamwera virus')

**E** ('vertical transmission'/exp OR 'vertical transmission') AND ('bunyamwera virus'/exp OR 'bunyamwera virus')

**E** ('blood donor'/exp OR 'blood donor') AND ('bunyamwera virus'/exp OR 'bunyamwera virus')

**Crimean Congo hemorrhagic fever virus**

**P** Transplantation AND 'Crimean Congo hemorrhagic fever virus

**P** Vertical transmission AND Crimean Congo hemorrhagic fever virus

**P** Blood donors AND Crimean Congo hemorrhagic fever virus

**E** ('transplantation'/exp OR transplantation) AND ('crimean-congo hemorrhagic fever virus'/exp OR 'crimean-congo hemorrhagic fever virus')

**E** ('vertical transmission'/exp OR 'vertical transmission') AND ('crimean-congo hemorrhagic fever virus'/exp OR 'crimean-congo hemorrhagic fever virus')

**E** ('blood donor'/exp OR 'blood donor') AND ('crimean-congo hemorrhagic fever virus'/exp OR 'crimean-congo hemorrhagic fever virus')

**Heartland virus**

**P** Transplantation AND Heartland virus

**P** Vertical transmission AND Heartland virus

**P** Blood donors AND Heartland virus

**E** ('transplantation'/exp OR transplantation) AND ('heartland virus'/exp OR 'heartland virus')

**E** ('vertical transmission'/exp OR 'vertical transmission') AND ('heartland virus'/exp OR 'heartland virus')

**E** ('blood donor'/exp OR 'blood donor') AND ('heartland virus'/exp OR 'heartland virus')

**Huaiyangshan banyangvirus (severe fever with thrombocytopenia syndrome virus)**

**P** Transplantation AND banyangvirus

**P** Vertical transmission AND banyangvirus

**P** Blood donors AND banyangvirus

**E** ('transplantation'/exp OR transplantation) AND ('severe fever with thrombocytopenia syndrome virus'/exp OR 'severe fever with thrombocytopenia syndrome virus')

**E** ('vertical transmission'/exp OR 'vertical transmission') AND ('severe fever with thrombocytopenia syndrome virus'/exp OR 'severe fever with thrombocytopenia syndrome virus')

**E** ('blood donor'/exp OR 'blood donor') AND ('severe fever with thrombocytopenia syndrome virus'/exp OR 'severe fever with thrombocytopenia syndrome virus')

**Jamestown Canyon virus**

**P** Transplantation AND Jamestown Canyon virus

**P** Vertical transmission AND Jamestown Canyon virus

**P** Blood donors AND Jamestown Canyon virus

**E** ('transplantation'/exp OR transplantation) AND ('jamestown canyon virus'/exp OR 'jamestown canyon virus')

**E** ('vertical transmission'/exp OR 'vertical transmission') AND ('jamestown canyon virus'/exp OR 'jamestown canyon virus')

**E** ('blood donor'/exp OR 'blood donor') AND ('jamestown canyon virus'/exp OR 'jamestown canyon virus')

**La Crosse virus**

**P** Transplantation AND La Crosse virus

**P** Vertical transmission AND La Crosse virus

**P** Blood donors AND La Crosse virus

**E** ('transplantation'/exp OR transplantation) AND ('la crosse virus'/exp OR 'la crosse virus')

**E** ('vertical transmission'/exp OR 'vertical transmission') AND ('la crosse virus'/exp OR 'la crosse virus')

**E** ('blood donor'/exp OR 'blood donor') AND ('la crosse virus'/exp OR 'la crosse virus')

**Rift Valley fever virus**

**P** Transplantation AND Rift Valley fever virus

**P** Vertical transmission AND Rift Valley fever virus

**P** Blood donors AND Rift Valley fever virus

**E** ('transplantation'/exp OR transplantation) AND ('rift valley fever virus'/exp OR 'rift valley fever virus')

**E** ('vertical transmission'/exp OR 'vertical transmission') AND ('rift valley fever virus'/exp OR 'rift valley fever virus')

**E** ('blood donor'/exp OR 'blood donor') AND ('rift valley fever virus'/exp OR 'rift valley fever virus')

**California enchephalitis virus (Tahyna virus)**

**P** Transplantation AND California encephalitis virus

**P** Vertical transmission AND California encephalitis virus

**P** Blood donors AND California encephalitis virus

**E** ('transplantation'/exp OR transplantation) AND ('california encephalitis virus'/exp OR 'california encephalitis virus')

**E** ('vertical transmission'/exp OR 'vertical transmission') AND ('california encephalitis virus'/exp OR 'california encephalitis virus')

**E** ('blood donor'/exp OR 'blood donor') AND ('california encephalitis virus'/exp OR 'california encephalitis virus')

**Toscana virus**

**P** Transplantation AND Toscana virus

**P** Vertical transmission AND Toscana virus

**P** Blood donors AND Toscana virus

**E** ('transplantation'/exp OR transplantation) AND ('toscana virus'/exp OR 'toscana virus')

**E** ('vertical transmission'/exp OR 'vertical transmission') AND ('toscana virus'/exp OR 'toscana virus')

**E** ('blood donor'/exp OR 'blood donor') AND ('toscana virus'/exp OR 'toscana virus')

**Dengue virus**

**P** Transplantation AND Dengue virus

**P** Vertical transmission AND Dengue virus

**P** Blood donors AND Dengue virus

**E** ('transplantation'/exp OR transplantation) AND ('dengue virus'/exp OR 'dengue virus')

**E** ('vertical transmission'/exp OR 'vertical transmission') AND ('dengue virus'/exp OR 'dengue virus')

**E** ('blood donor'/exp OR 'blood donor') AND ('dengue virus'/exp OR 'dengue virus')

**Japanese encephalitis virus**

**P** Transplantation AND Japanese encephalitis virus

**P** Vertical transmission AND Japanese encephalitis virus

**P** Blood donors AND Japanese encephalitis virus

**E** ('transplantation'/exp OR transplantation) AND ('japanese encephalitis virus'/exp OR 'japanese encephalitis virus')

**E** ('vertical transmission'/exp OR 'vertical transmission') AND ('japanese encephalitis virus'/exp OR 'japanese encephalitis virus')

**E** ('blood donor'/exp OR 'blood donor') AND ('japanese encephalitis virus'/exp OR 'japanese encephalitis virus')

**Kyasanur forest disease virus**

**P** Transplantation AND Kyasanur forest disease virus

**P** Vertical transmission AND Kyasanur forest disease virus

**P** Blood donors AND Kyasanur forest disease virus

**E** ('transplantation'/exp OR transplantation) AND ('kyasanur forest disease virus'/exp OR 'kyasanur forest disease virus')

**E** ('vertical transmission'/exp OR 'vertical transmission') AND ('kyasanur forest disease virus'/exp OR 'kyasanur forest disease virus')

**E** ('blood donor'/exp OR 'blood donor') AND ('kyasanur forest disease virus'/exp OR 'kyasanur forest disease virus')

**Louping ill virus**

**P** Transplantation AND Louping ill virus

**P** Vertical transmission AND Louping ill virus

**P** Blood donors AND Louping ill virus

**E** ('transplantation'/exp OR transplantation) AND ('louping ill virus'/exp OR 'louping ill virus')

**E** ('vertical transmission'/exp OR 'vertical transmission') AND ('louping ill virus'/exp OR 'louping ill virus')

**E** ('blood donor'/exp OR 'blood donor') AND ('louping ill virus'/exp OR 'louping ill virus')

**Murray valley encephalitis virus
P** Transplantation AND Murray valley encephalitis virus

**P** Vertical transmission AND Murray valley encephalitis virus

**P** Blood donors AND Murray valley encephalitis virus

**E** ('transplantation'/exp OR transplantation) AND ('murray valley encephalitis virus'/exp OR 'murray valley encephalitis virus')

**E** ('vertical transmission'/exp OR 'vertical transmission') AND ('murray valley encephalitis virus'/exp OR 'murray valley encephalitis virus')

**E** ('blood donor'/exp OR 'blood donor') AND ('murray valley encephalitis virus'/exp OR 'murray valley encephalitis virus')

**Spondweni virus**

**P** Transplantation AND Spondweni virus

**P** Vertical transmission AND Spondweni virus

**P** Blood donors AND Spondweni virus

**E** ('transplantation'/exp OR transplantation) AND ('spondweni virus'/exp OR 'spondweni virus')

**E** ('vertical transmission'/exp OR 'vertical transmission') AND ('spondweni virus'/exp OR 'spondweni virus')

**E** ('blood donor'/exp OR 'blood donor') AND ('spondweni virus'/exp OR 'spondweni virus')

**St. Louis encephalitis virus**

**P** Transplantation AND St. Louis encephalitis virus

**P** Vertical transmission AND St. Louis encephalitis virus

**P** Blood donors AND St. Louis encephalitis virus

**E** ('transplantation'/exp OR transplantation) AND ('st. louis encephalitis virus'/exp OR 'st. louis encephalitis virus')

**E** ('vertical transmission'/exp OR 'vertical transmission') AND ('st. louis encephalitis virus'/exp OR 'st. louis encephalitis virus')

**E** ('blood donor'/exp OR 'blood donor') AND ('st. louis encephalitis virus'/exp OR 'st. louis encephalitis virus')

**Tick-borne encephalitis virus**

**P** Transplantation AND Tick-borne encephalitis virus

**P** Vertical transmission AND Tick-borne encephalitis virus

**P** Blood donors AND Tick-borne encephalitis virus

**E** ('transplantation'/exp OR transplantation) AND ('tick borne encephalitis virus'/exp OR 'tick borne encephalitis virus')

**E** ('vertical transmission'/exp OR 'vertical transmission') AND ('tick borne encephalitis virus'/exp OR 'tick borne encephalitis virus')

**E** ('blood donor'/exp OR 'blood donor') AND ('tick borne encephalitis virus'/exp OR 'tick borne encephalitis virus')

**West Nile virus**

**P** Transplantation AND West Nile virus

**P** Vertical transmission AND West Nile virus

**P** Blood donors AND West Nile virus

**E** ('transplantation'/exp OR transplantation) AND ('west nile virus'/exp OR 'west nile virus')

**E** ('vertical transmission'/exp OR 'vertical transmission') AND ('west nile virus'/exp OR 'west nile virus')

**E** ('blood donor'/exp OR 'blood donor') AND ('west nile virus'/exp OR 'west nile virus')

**Yellow fever virus**

**P** Transplantation AND Yellow fever virus

**P** Vertical transmission AND Yellow fever virus

**P** Blood donors AND Yellow fever virus

**E** ('transplantation'/exp OR transplantation) AND ('yellow fever virus'/exp OR 'yellow fever virus')

**E** ('vertical transmission'/exp OR 'vertical transmission') AND ('yellow fever virus'/exp OR 'yellow fever virus')

**E** ('blood donor'/exp OR 'blood donor') AND ('yellow fever virus'/exp OR 'yellow fever virus')

**Zika virus**

**P** Transplantation AND Zika virus

**P** Vertical transmission AND Zika virus

**P** Blood donors AND Zika virus

**E** ('transplantation'/exp OR transplantation) AND ('zika virus'/exp OR 'zika virus')

**E** ('vertical transmission'/exp OR 'vertical transmission') AND ('zika virus'/exp OR 'zika virus')

**E** ('blood donor'/exp OR 'blood donor') AND ('zika virus'/exp OR 'zika virus')

**Banna virus**

**P** Transplantation AND Banna virus

**P** Vertical transmission AND Banna virus

**P** Blood donors AND Banna virus

**E** ('transplantation'/exp OR transplantation) AND ('banna virus'/exp OR 'banna virus')

**E** ('vertical transmission'/exp OR 'vertical transmission') AND ('banna virus'/exp OR 'banna virus')

**E** ('blood donor'/exp OR 'blood donor') AND ('banna virus'/exp OR 'banna virus')

**Bluetongue virus**

**P** Transplantation AND Bluetongue virus

**P** Vertical transmission AND Bluetongue virus

**P** Blood donors AND Bluetongue virus

**E** ('transplantation'/exp OR transplantation) AND ('bluetongue orbivirus'/exp OR 'bluetongue orbivirus')

**E** ('vertical transmission'/exp OR 'vertical transmission') AND ('bluetongue orbivirus'/exp OR 'bluetongue orbivirus')

**E** ('blood donor'/exp OR 'blood donor') AND ('bluetongue orbivirus'/exp OR 'bluetongue orbivirus')

**Colorado tick fever virus**

**P** Transplantation AND Colorado tick fever virus

**P** Vertical transmission AND Colorado tick fever virus

**P** Blood donors AND Colorado tick fever virus

**E** ('transplantation'/exp OR transplantation) AND ('colorado tick fever virus'/exp OR 'colorado tick fever virus')

**E** ('vertical transmission'/exp OR 'vertical transmission') AND ('colorado tick fever virus'/exp OR 'colorado tick fever virus')

**E** ('blood donor'/exp OR 'blood donor') AND ('colorado tick fever virus'/exp OR 'colorado tick fever virus')

**Vesicular stomatitis virus (Vesiculovirus Piry)**

**P** Transplantation AND Vesicular stomatitis virus

**P** Vertical transmission AND Vesicular stomatitis virus

**P** Blood donors AND Vesicular stomatitis virus

**E** ('transplantation'/exp OR transplantation) AND ('vesicular stomatitis indiana virus'/exp OR 'vesicular stomatitis indiana virus')

**E** ('vertical transmission'/exp OR 'vertical transmission') AND ('vesicular stomatitis indiana virus'/exp OR 'vesicular stomatitis indiana virus')

**E** ('blood donor'/exp OR 'blood donor') AND ('vesicular stomatitis indiana virus'/exp OR 'vesicular stomatitis indiana virus')

**Barmah forest virus**

**P** Transplantation AND Barmah forest virus

**P** Vertical transmission AND Barmah forest virus

**P** Blood donors AND Barmah forest virus

**E** ('transplantation'/exp OR transplantation) AND ('barmah forest virus'/exp OR 'barmah forest virus')

**E** ('vertical transmission'/exp OR 'vertical transmission') AND ('barmah forest virus'/exp OR 'barmah forest virus')

**E** ('blood donor'/exp OR 'blood donor') AND ('barmah forest virus'/exp OR 'barmah forest virus')

**Chikungunya virus**

**P** Transplantation AND Chikungunya virus

**P** Vertical transmission AND Chikungunya virus

**P** Blood donors AND Chikungunya virus

**E** ('transplantation'/exp OR transplantation) AND ('chikungunya virus'/exp OR 'chikungunya virus')

**E** ('vertical transmission'/exp OR 'vertical transmission') AND ('chikungunya virus'/exp OR 'chikungunya virus')

**E** ('blood donor'/exp OR 'blood donor') AND ('chikungunya virus'/exp OR 'chikungunya virus')

**Eastern equine encephalitis virus**

**P** Transplantation AND Eastern equine encephalitis virus

**P** Vertical transmission AND Eastern equine encephalitis virus

**P** Blood donors AND Eastern equine encephalitis virus

**E** ('transplantation'/exp OR transplantation) AND ('eastern equine encephalitis virus'/exp OR 'eastern equine encephalitis virus')

**E** ('vertical transmission'/exp OR 'vertical transmission') AND ('eastern equine encephalitis virus'/exp OR 'eastern equine encephalitis virus')

**E** ('blood donor'/exp OR 'blood donor') AND ('eastern equine encephalitis virus'/exp OR 'eastern equine encephalitis virus')

**Mayaro virus**

**P** Transplantation AND Mayaro virus

**P** Vertical transmission AND Mayaro virus

**P** Blood donors AND Mayaro virus

**E** ('transplantation'/exp OR transplantation) AND ('mayaro virus'/exp OR 'mayaro virus') 0

**E** ('vertical transmission'/exp OR 'vertical transmission') AND ('mayaro virus'/exp OR 'mayaro virus')

**E** ('blood donors'/exp OR 'blood donors') AND ('mayaro virus'/exp OR 'mayaro virus')

**O’nyong o’nyong virus**

**P** Transplantation AND O’nyong o’nyong virus

**P** Vertical transmission AND O’nyong o’nyong virus

**P** Blood donors AND O’nyong o’nyong virus

**E** ('transplantation'/exp OR transplantation) AND ('o nyong nyong virus'/exp OR 'o nyong nyong virus')

**E** ('vertical transmission'/exp OR 'vertical transmission') AND ('o nyong nyong virus'/exp OR 'o nyong nyong virus')

**E** ('blood donor'/exp OR 'blood donor') AND ('o nyong nyong virus'/exp OR 'o nyong nyong virus')

**Ross river virus**

**P** Transplantation AND Ross river virus

**P** Vertical transmission AND Ross river virus

**P** Blood donors AND Ross river virus

**E** ('transplantation'/exp OR transplantation) AND ('ross river virus'/exp OR 'ross river virus')

**E** ('vertical transmission'/exp OR 'vertical transmission') AND ('ross river virus'/exp OR 'ross river virus')

**E** ('blood donor'/exp OR 'blood donor') AND ('ross river virus'/exp OR 'ross river virus')

**Sindbis virus**

**P** Transplantation AND Sindbis virus

**P** Vertical transmission AND Sindbis virus

**P** Blood donors AND Sindbis virus

**E** ('transplantation'/exp OR transplantation) AND ('sindbis virus'/exp OR 'sindbis virus')

**E** ('vertical transmission'/exp OR 'vertical transmission') AND ('sindbis virus'/exp OR 'sindbis virus')

**E** ('blood donor'/exp OR 'blood donor') AND ('sindbis virus'/exp OR 'sindbis virus')

**Venezuelan equine encephalitis virus**

**P** Transplantation AND Venezuelan equine encephalitis virus

**P** Vertical transmission AND Venezuelan equine encephalitis virus

**P** Blood donors AND Venezuelan equine encephalitis virus

**E** ('transplantation'/exp OR transplantation) AND ('venezuelan equine encephalitis virus'/exp OR 'venezuelan equine encephalitis virus')

**E** ('vertical transmission'/exp OR 'vertical transmission') AND ('venezuelan equine encephalitis virus'/exp OR 'venezuelan equine encephalitis virus')

**E** ('blood donor'/exp OR 'blood donor') AND ('venezuelan equine encephalitis virus'/exp OR 'venezuelan equine encephalitis virus')

**Western equine encephalitis virus**

**P** Transplantation AND Western equine encephalitis virus

**P** Vertical transmission AND Western equine encephalitis virus

**P** Blood donors AND Western equine encephalitis virus

**E** ('transplantation'/exp OR transplantation) AND ('western equine encephalitis virus'/exp OR 'western equine encephalitis virus')

**E** ('vertical transmission'/exp OR 'vertical transmission') AND ('western equine encephalitis virus'/exp OR 'western equine encephalitis virus')

**E** ('blood donor'/exp OR 'blood donor') AND ('western equine encephalitis virus'/exp OR 'western equine encephalitis virus')
