# Supplementary material for: Transfusion-transmitted arboviruses: Update and systematic review
Source: PLoS Negl Trop Dis. 2022 Oct 6;16(10):e0010843. doi: 10.1371/journal.pntd.0010843 (PMC9578600; doi:10.1371/journal.pntd.0010843)
Supplement: S1 Flow Chart — (DOCX) [file pntd.0010843.s004.docx]

| **S1 Flow Chart**. PRISMA flow chart on selection of case studies of transfusion-transmitted arboviruses | | | | | | |
| --- | --- | --- | --- | --- | --- | --- |
| **Viruses** | | **Human disease** | **Total records** | **Records after duplicates removed** | **Full-text** | **Selected** |
| ***Asfarviridae* family (Baltimore group I)** | |  |  |  |  |  |
|  | African Swine Fever Virus | No |  | | | |
| ***Bunyaviridae* family (Baltimore group V)** | |  |  |  |  |  |
|  | Bunyamwera virus | Yes | − | − | − | − |
|  | Crimean Congo hemorrhagic fever virus | Yes | 67 | 41 | 8 | − |
|  | Heartland virus | Yes | 5 | 5 | − | − |
|  | Huaiyangshan banyangvirus (SFTS virus) | Yes | − | − | − | − |
|  | Jamestown Canyon virus | Yes | − | − | − | − |
|  | La Crosse virus | Yes | − | − | − | − |
|  | Rift Valley fever virus | Yes | 13 | 8 | 1 | − |
|  | Tahyana orthobunyavirus | Yes | − | − | − | − |
|  | Toscana virus | Yes | 8 | 8 | − | − |
| ***Flaviviridae* family (Baltimore group IV)** | |  |  |  |  |  |
|  | Bagaza virus | No |  |  |  |  |
|  | Dengue virus | Yes | 1313 | 764 | 46 | 9 |
|  | Entebbe bat virus | No |  | | | |
|  | Japanese encephalitis virus | Yes | 106 | 60 | 2 | 1 |
|  | Kyasanur Forest disease virus | Yes | 12 | 12 | 1 | − |
|  | Langat virus | No |  | | | |
|  | Louping ill virus | Yes | 12 | 12 | 1 | − |
|  | Murray Valley encephalitis virus | Yes | 5 | 5 | − | − |
|  | Sokoluk virus | No |  | | | |
|  | Spondweni virus | Yes | − | − | − | − |
|  | St. Louis encephalitis virus | Yes | 21 | 12 | 2 | 1 |
|  | Tamana bat virus | No |  | | | |
|  | Tick-borne encephalitis virus | Yes | 67 | 44 | 7 | 1 |
|  | Powassan virus | Yes | 32 | 21 | 4 | 1 |
|  | West Nile fever virus | Yes | 1487 | 778 | 60 | 11 |
|  | Yellow fever virus | Yes | 91 | 60 | 2 | 1 |
|  | Yokose virus | No |  | | | |
|  | Zika virus | Yes | 993 | 536 | 32 | 2 |
| ***Reoviridae* family (Baltimore group III)** | |  |  |  |  |  |
|  | African horse sickness virus | No |  | | | |
|  | Banna virus | Yes | − | − | − | − |
|  | Bluetongue virus | No |  |  |  |  |
|  | Colorado tick fever virus | Yes | 5 | 3 | 3 | 1 |
|  | Epizootic hemorrhagic disease virus | No |  | | | |
|  | Equine encephalosis viruss | No |  | | | |
|  | Middle point orbivirus | No |  | | | |
| ***Togaviridae* family (Baltimore group IV)** | |  |  |  |  |  |
|  | Barmah forest virus | Yes | 110 | 73 | − | − |
|  | Buggy creek virus | No |  | | | |
|  | Chikungunya virus | Yes | 419 | 245 | 19 | − |
|  | Eastern equine encephalitis virus | Yes | 5 | 5 | 2 | − |
|  | Mayaro virus | Yes | 13 | 8 | 1 | − |
|  | Ndumu virus | No |  | | | |
|  | O’nyong’nyong virus | Yes | − | − | − | − |
|  | Ross River virus | Yes | 66 | 30 | 7 | 1 |
|  | Sindbis virus | Yes | 98 | 49 | 1 | − |
|  | Venezuelan equine encephalitis virus | Yes | − | − | − | − |
|  | Western equine encephalitis virus | Yes | − | − | − | − |
| ***Rhabdoviridae* family (Baltimore group V)** | |  |  |  |  |  |
|  | Bovine ephemeral fever virus | No |  |  |  |  |
|  | Vesiculovirus Piry (Vesicular stomatitis virus) | Yes | 210 | 139 | − | − |
