# Supplementary material for: Transfusion-transmitted arboviruses: Update and systematic review
Source: PLoS Negl Trop Dis. 2022 Oct 6;16(10):e0010843. doi: 10.1371/journal.pntd.0010843 (PMC9578600; doi:10.1371/journal.pntd.0010843)
Supplement: S2 Flow Chart — (DOCX) [file pntd.0010843.s005.docx]

| **Table S5.1**  Flow chart for the process of selecting records describing other mechanisms of direct transmission (non-vectorial transmission) of different arboviruses and publications reporting the prevalence of arboviruses in blood donors. | | | | | | | | | | |
| --- | --- | --- | --- | --- | --- | --- | --- | --- | --- | --- |
| **Viruses** | | **Screening in blood donors** | | | **Transplantation** | | | **Vertical transmission** | | |
|  |  | **Total records** | **Pre-selected** | **Included** | **Total records** | **Pre-selected** | **Indluced** | **Total records** | **Pre-selected** | **Included** |
| ***Asfarviridae* family (Baltimore group I)** | |  |  |  |  |  |  |  |  |  |
|  | African Swine Fever Virus |  |  | |  | |  |  |  |  |
| ***Bunyaviridae* family (Baltimore group V)** | |  |  |  |  |  |  |  |  |  |
|  | Bunyamwera virus | − | − | − | 3 | − | − | − | − | − |
|  | Crimean Congo hemorrhagic fever virus | 18 | 2 | 1 | 4 | − | − | 1 | 1 | 1 |
|  | Heartland virus | − | − | − | 30 | − | − | − | − | − |
|  | Huaiyangshan banyangvirus (SFTS) | 4 | 2 | 1 | − | − | − | − | − | − |
|  | Jamestown Canyon virus | 12 | 2 | 1 | 23 | 1 | 1 | − | − | − |
|  | La Crosse virus | 2 | − | − | 7 | − | − | 2 | 2 | 1 |
|  | Rift Valley fever virus | 1 | 1 | 1 | 10 | − | − | 55 | 2 | 1 |
|  | Tahyana orthobunyavirus | 13 | 1 | 1 | 26 | − | − | 27 | − | − |
|  | Toscana virus | 15 | − | 1 | 15 | − | − | 6 | − | − |
| ***Flaviviridae* family (Baltimore group IV)** | |  |  |  |  |  |  |  |  |  |
|  | Dengue virus | 440 | 63 | 1 | 373 | 6 | 1 | 451 | 20 | 1 |
|  | Japanese encephalitis virus | 39 | 2 | 1 | 82 | − | − | 58 | 1 | 1 |
|  | Kyasanur Forest disease virus |  |  |  |  |  |  |  |  |  |
|  | Louping ill virus |  |  |  |  |  |  |  |  |  |
|  | Murray Valley encephalitis virus | 9 | 2 | 1 | 3 | − | − | 5 | − | − |
|  | Spondweni virus | − | − | − | − | − | − | 2 | − | − |
|  | St. Louis encephalitis virus | 12 | 1 | 1 | 12 | − | − | 24 | − | − |
|  | Tick-borne encephalitis virus | 74 | 5 | 1 | 40 | − | − | 26 | − | − |
|  | Powassan virus |  |  |  |  |  |  |  |  |  |
|  | West Nile fever virus | 767 | 60 | 1 | 618 | 17 | 1 | 173 | 2 | 1 |
|  | Yellow fever virus | 51 | 1 | 1 | 77 | − | − | 85 | 1 | 1 |
|  | Zika virus | 317 | 34 | 1 | 233 | − | − | 835 | 18 | 1 |
| ***Reoviridae* family (Baltimore group III)** | |  |  |  |  |  |  |  |  |  |
|  | Banna virus | 1 | − | − | 7 | − | − | − | − | − |
|  | Bluetongue virus | 5 | − | − | 7 | − | − | 73 | − | − |
|  | Colorado tick fever virus | 3 | − | − | − | − | − | 1 | 1 | 1 |
| ***Rhabdoviridae* family (Baltimore group V)** | |  |  |  |  |  |  |  |  |  |
|  | Piry vesiculovirus (Vesicular stomatitis virus) | 1 | 1 | 1 | 198 | − | − | 13 | − | − |
| ***Togaviridae* family (Baltimore group IV)** | |  |  |  |  |  |  |  |  |  |
|  | Barmah forest virus | 59 | 1 | 1 | 100 | − | − | 100 | − | − |
|  | Chikungunya virus | 161 | 17 | 1 | 113 | − | − | 223 | 18 | 1 |
|  | Eastern equine encephalitis virus | 4 | 1 | 1 | 6 | 1 | 1 | 6 | − | − |
|  | Mayaro virus | 5 | 1 | 1 | 3 | − | − | 7 | − | − |
|  | O'nyong'nyong virus | 1 | 1 | 1 | − | − | − | 1 | 1 | 1 |
|  | Ross River virus | 29 | 6 | 1 | 6 | − | − | 13 | − | 1 |
|  | Sindbis virus | 1 | 1 | 1 | 18 | − | − | 5 | 1 | 1 |
|  | Venezuelan equine encephalitis virus | − | − | − | 20 | − | − | 5 | − | 1 |
|  | Western equine encephalitis virus | 1 | − | − | − | − | − | 5 | 1 | 1 |
